# Synthesis, Characterization, Antioxidant, and Anticancer Activity against Colon Cancer Cells of Some Cinnamaldehyde-Based Chalcone Derivatives

**DOI:** 10.3390/biom14020216

**Published:** 2024-02-12

**Authors:** Mohamed A. El-Atawy, Demiana H. Hanna, Ali H. Bashal, Hoda A. Ahmed, Eida M. Alshammari, Ezzat A. Hamed, Abdullah R. Aljohani, Alaa Z. Omar

**Affiliations:** 1 Department of Chemistry, College of Science in Yanbu, Taibah University, Yanbu Governorate 30799, Saudi Arabia; maatawy@taibahu.edu.sa (M.A.E.-A.); abishil@taibahu.edu.sa (A.H.B.); hmahmoud@taibahu.edu.sa (H.A.A.); abdullah.rajaallah@gmail.com (A.R.A.); 2 Chemistry Department, Faculty of Science, Alexandria University, P.O. Box 426 Ibrahemia, Alexandria 21321, Egypt; ezzat.awad@alexu.edu.eg (E.A.H.); alaazaki@alexu.edu.eg (A.Z.O.); 3 Department of Chemistry, Faculty of Science, Cairo University, Giza 12613, Egypt; 4 Department of Chemistry, College of Sciences, University of Ha’il, Ha’il 55473, Saudi Arabia; eida.alshammari@uoh.edu.sa; 5 Saudi Irrigation Organization (SIO), Al-Hassa 31982, Saudi Arabia

**Keywords:** chalcone derivatives, antioxidant, colon cancer, cytotoxicity, apoptosis

## Abstract

The purpose of the current investigation was to produce cinammaldehyde-based chalcone derivatives (**3a**–**k**) to evaluate their potential effectiveness as antioxidant and inhibitory agents versus human Caco-2 cancer cells. The findings obtained using the DPPH assay showed that compound **3e** had the highest effective antioxidant activity with the best IC_50_ value compared with the other compounds. Moreover, the cytotoxic findings revealed that compound **3e** was the best compound for inhibiting Caco-2 development in contrast to all other produced derivatives, with the lowest IC_50_ concentration (32.19 ± 3.92 µM), and it also had no detrimental effects on healthy human lung cells (wi38 cells). Exposure of Caco-2 cells with this IC_50_ value of compound **3e** resulted in a substantial rise in the number of early and late cells that are apoptotic with a significant comet nucleus when compared with control cells employing the annexin V/PI and comet evaluations, respectively. Furthermore, qRT-PCR and ELISA examinations indicated that compound **3e** significantly altered the expression of genes and their relative proteins related to apoptosis in the treated Caco-2 cells, thus significantly inhibiting Caco-2 growth through activating Caspase-3 via an intrinsic apoptotic pathway. As a result, compound **3e** could serve as an effective therapy for human colon cancer.

## 1. Introduction

Chalcones have gained significant attention in recent years due to their possible application in the treatment of numerous cancers, including colon cancer [1,2,3,4,5,6,7,8,9]. Colon cancer is among the most common malignancies worldwide, and finding effective treatments is of utmost importance [10,11,12]. Several studies have demonstrated the promising anti-cancer properties of chalcones in colon cancer treatment through induction of apoptosis [5,13,14,15] in colon cancer cells by regulating multiple signaling pathways involved in cell growth and survival.

One of the key mechanisms through which chalcones exert their anti-cancer effects is by inhibiting the growth and proliferation of cancer cells [16]. It is able to do this by interfering with the cell cycle progression, specifically targeting the G2/M phase [17,18], which is crucial for cancer cell division. By blocking this phase, chalcones effectively prevent the replication and growth of colon cancer cells. Moreover, chalcones have been found to possess potent anti-inflammatory properties [19,20,21,22,23]. Chronic inflammation is frequently linked to the onset and spread of colon cancer. By reducing the levels of inflammatory molecules and inhibiting the activation of inflammatory signaling pathways, chalcones suppress the pro-inflammatory environment that promotes cancer growth.

Furthermore, chalcones exhibit strong antioxidant activity [24,25,26,27], which helps combat oxidative stress in colon cancer cells. Oxidative stress is a major contributor to cancer initiation and progression, and chalcone’s ability to neutralize free radicals and enhance the cellular antioxidant defense system helps protect against DNA damage and inhibit tumor growth. Additionally, chalcones have been reported to inhibit angiogenesis [28,29,30,31], the formation of new blood vessels that supply nutrients and oxygen to tumors. By disrupting the process of angiogenesis, chalcones effectively starve the cancer cells, hindering their growth and metastasis.

Heterocyclic compounds incorporating pyridine, furan, thiophene, or pyrrole moieties have long represented privileged structures in anticancer drug discovery [32,33]. Pyridine derivatives can have diverse functions, including enzyme inhibition, receptor binding, and DNA intercalation, making them useful for targeting cancer cells [34,35]. Furan derivatives in anticancer drugs can have various mechanisms of action, such as inhibiting specific enzymes or interfering with DNA replication. One example of a furan-containing anticancer drug is lapatinib.

Thiophene-containing compounds often exhibit anticancer effects by targeting specific receptors or enzymes involved in cancer progression [36]. For instance, the drug Raloxifene contains a thiophene moiety is employed in the therapeutic management of breast cancer. Pyrrole derivatives are found in many anticancer drugs and can inhibit enzymes [37], interact with receptors, or act as DNA alkylating agents. One example of a pyrrole-containing anticancer drug is Sunitinib, which is used to treat renal cell carcinoma and imatinib-resistant gastrointestinal stromal tumors. Moreover, these selected heterocyclic rings include electron-deficient rings such as pyridine and electron-rich rings such as furan, thiophene, and pyrrole. Moreover, pyridine possess basic characteristics, but on the other hand, pyrrole has certain acidic properties and both of them are capable for intermolecular hydrogen bonding. This explains the variety of their properties and the different pathways for their potential interactions with biological targets.

For these reasons, the current research aims to develop hybrid pharmacophores based on chalcones derived from a naturally occurring cinnamaldehyde and the above-mentioned heterocyclic rings to assess their efficacy as antioxidant and anticancer agents against Caco-2 cancer cells while elucidating their cytotoxic mechanisms.

## 2. Results and Discussion

### 2.1. Chemistry

**Scheme 1:**
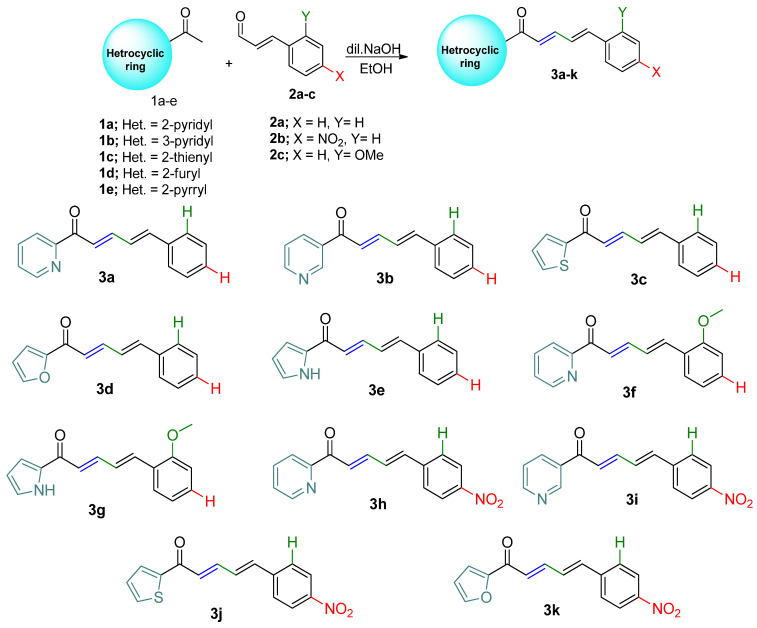
Synthesis of chalcones **3a**–**k**.

Synthesis of cinammaldehyde-based chalcone derivatives **3a**–**k** was performed as outlined in Scheme 1. A Claisen–Schmidt condensation reaction between equimolar amounts of methyl heteroarylketone **1a**–**e** as the nucleophilic component with Cinnamaldehyde **2a**, 4-nitrocinnamaldehyde **2b**, or 2-methoxycinnamaldehyde **2c** as the electrophilic component has been carried out at room temperature in the presence of aqueous sodium hydroxide in ethanol and under magnetic stirring for three hours to yield the corresponding chalcones **3a**–**k**. All the prepared chalcones were water insoluble, but soluble in most organic solvents. The chalcones **3a**–**k** were recrystallized from pure ethanol and isolated as yellow solids. The structures of our synthesized chalcones were confirmed using proton NMR and carbon-13 NMR (see Supplementary File S1).

The nitro group has been incorporated in the structure of chalcones **3h**–**k** to enhance the electrophilicity and chemical reactivity for these compounds. Moreover, compounds containing nitro group would show different biological interactions involving the biotransformation of the nitro group, releasing intermediates in the redox process and causing changes in the stability of membrane structures of several cells [38].

### 2.2. Antioxidant Effects

**Figure 1:**
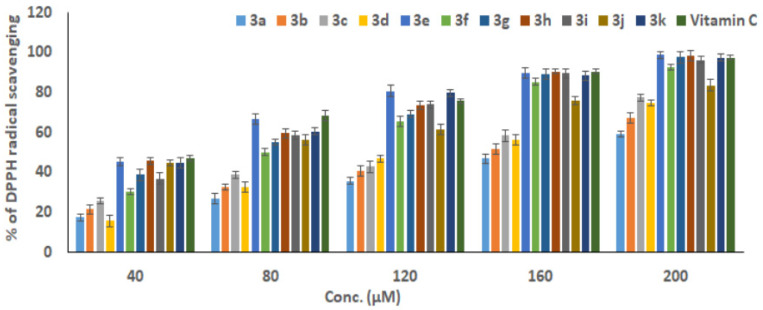
The studied compounds’ antioxidant capabilities in relation to vitamin C against DPPH radicals at various dosages. The results of three different analyses are displayed as the mean ± SD.

**Figure 2:**
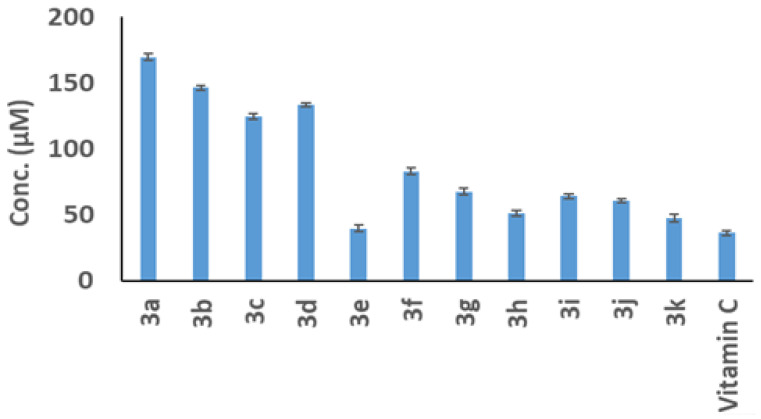
The scavenging activity of the tested compounds for DPPH radicals as measured using the IC_50_ concentration in relation to the reference ascorbic acid. The results of three distinct analyses are represented as the mean ± SD of the values.

Free-radical DPPH inhibitory testing represents one of the most frequently employed techniques to gauge the antioxidant abilities of each tested compound. Consequentially, the current investigation evaluated the examined compounds’ antioxidant activity with regard to their capability to scavenge DPPH. The capacities of the compounds being studied to scavenge free radicals were assessed at various concentrations (40–200 µM) versus the standard ascorbic acid (Figure 1). For all compounds, the scavenging activity % for DPPH was increased when increasing the concentration up to 200 µM. Additionally, Figure 2 shows the scavenging activity for DPPH for all examined compounds as measured in terms of IC_50_ in contrast to the reference ascorbic acid. The obtained findings showed that the **3e** compound had the highest effective antioxidant activity with the best IC_50_ value compared with the other compounds.

### 2.3. Cytotoxicity Evaluations

**Figure 3:**
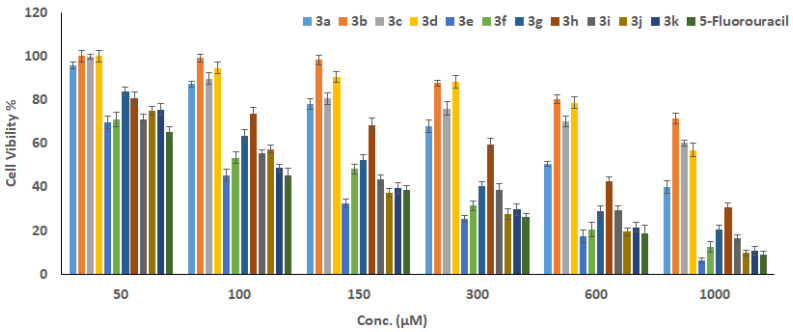
The viability percentage of the tested compounds against Caco-2 cells using the MTT technique compared to the 5-Fluorouracil reference drug. The results of three distinct analyses are represented as the mean ± SD for the values.

**Figure 4:**
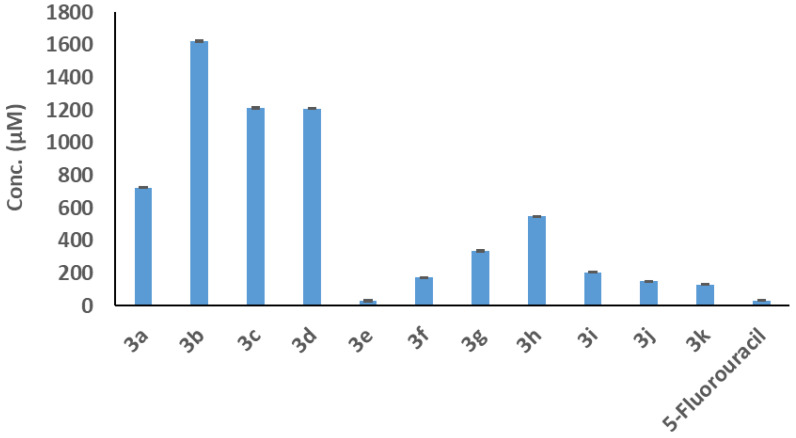
The IC_50_ values of the studied compounds against Caco-2 cells were compared to the IC_50_ value of the 5-Fluorouracil reference drug using the MTT procedure. The results of three distinct analyses are represented as the mean ± SD for the values.

The harmful effects of the tested compounds (**3a**–**3k**) and the 5-Fluorouracil standard drug on the development of Caco-2 cells were evaluated using the MTT method. As shown in Figure 3, increasing the dosages from 50 to 1000 µM for 24 h of incubation resulted in a decrease in the percentage of viable Caco-2 cells for all the compounds examined. The obtained IC_50_ values for compounds **3a**, **3b**, **3c**, **3d**, **3e**, **3f**, **3g**, **3h**, **3i**, **3j**, and **3k** compared to the IC_50_ value of the 5-Fluorouracil (5-FU) drug are displayed in Figure 4 and Table 1. These data were calculated based on the associations between compound dosages and their impact on Caco-2 cell viability. This finding implies that compound **3e**, which possesses the most effective IC_50_ concentration (32.19 ± 3.9 µM) among other compounds, may have a stronger toxic effect on Caco-2 cell proliferation. Moreover, there was non-significant variation between the IC_50_ value of compound **3e** (32.1 ± 3.92 µM) and the IC_50_ value of 5-the Fluorouracil reference drug (33.12 ± 1.45 µM).

**Figure 5:**
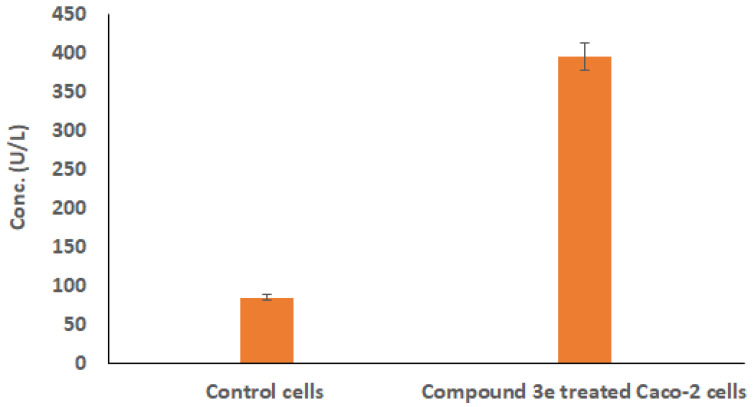
The levels of LDH enzyme in compound 3e-treated Caco-2 cells to those in untreated ones using ELISA test. The results of three distinct analyses are represented as mean ± SD for the values.

To provide additional evidence that compound **3e** inhibits the growth of Caco-2 cells, the lactate dehydrogenase enzyme’s activity was tested experimentally. The outcomes demonstrated that LDH levels in compound **3e**-treated Caco-2 cells considerably increased in comparison to levels in untreated ones (Figure 5), *p* < 0.001. Therefore, the outcomes of the LDH and MTT examinations pointed to compound **3e**’s strong effectiveness against the growth of the tested Caco-2 cells.

**Figure 6:**
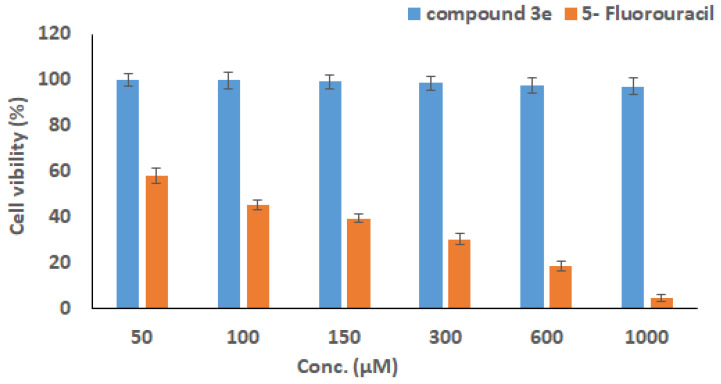
The correlation between the viability of normal cells (wi38) following treatment with various doses of the compound **3e** and the 5-Fluorouracil reference drug. The MTT test was used to correlate the survival of normal cells (wi38) upon treatment at various doses of the chemical **3e** and 5-Fluorouracil drug (50–1000 µM) over 24 h. The findings are presented as the mean ± SD of three separate assessments.

In addition, upon exposure of wi38 cells from human lung normal tissue to dosages of compound **3e** and the 5-Fluorouracil drug in the range of 50 to 1000 µM, higher % of wi38 cell viability was detected after treatment with compound **3e** through all its tested concentrations (Figure 6). These results showed that compound **3e**, although showing safety and biocompatibility traits with regard to healthy cells, demonstrated specific toxicity towards cancer cells. However, the 5-Fluorouracil drug showed a decrease in wi38 cell viability with increasing its concentration (Figure 6), where the obtained IC_50_ value against normal wi38 cell (22.79 ± 2.46 µM) was significantly lower than the IC_50_ value against the Caco-2 cancer cell (33.12 ± 1.45 µM), clarifying that the anticancer doses of 5-FU are toxic to normal cells.

### 2.4. Quantification of DNA Damage and Apoptosis in Caco-2 Cells

**Figure 7:**
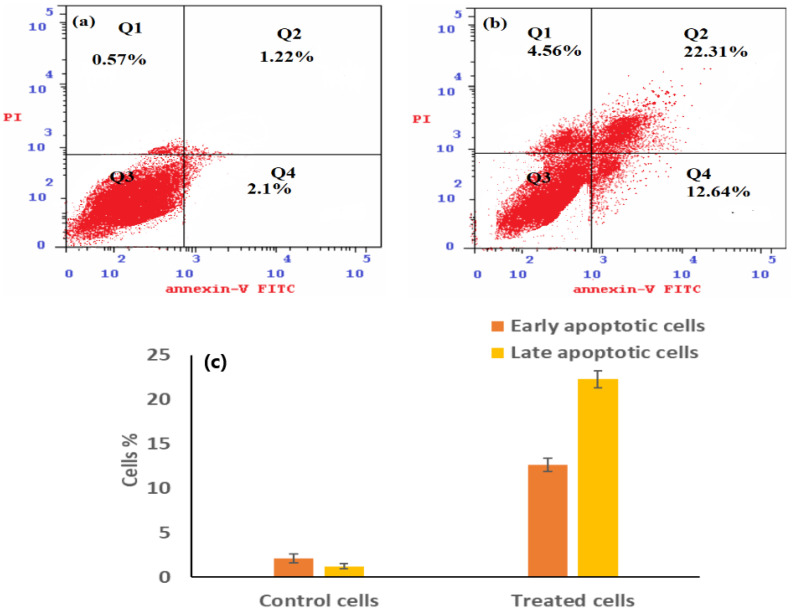
Employing flow cytometry, the apoptotic behavior of Caco-2 cells was observed, illustrating necrotic cells (Q1: An−, PI+), late apoptotic cells (Q2: An+, PI+), viable cells (Q3: An−, PI−), and early apoptotic cells (Q4: An+, PI−) for treated Caco-2 cells using an IC_50_ dose of compound **3e** (**b**) compared to Caco-2 cancer cells that have not been treated (**a**) using flow cytometry. A histogram (**c**) depicts the proportion of early and late apoptotic cells in compound **3e**-treated cells relative to untreated cells. Three different findings are shown as mean ± SD, *p* < 0.001.

Compound **3e**’s capability was evaluated using comet and annexin V/PI evaluations to trigger damage to DNA and apoptosis in Caco-2 cells, respectively. Figure 7a,b shows the results of flow cytometry analysis for untreated cells in comparison to cells treated with compound **3e**, respectively, using annexin V/PI labeling. In contrast to untreated Caco-2 cells, compound **3e**-treated Caco-2 cells were shown to have a much greater percentage of apoptotic cells (encompassing the two early and late stages). Further, comparing treated cells with compound **3e** to untreated cells, Figure 7c demonstrates the number of early and late cells that are apoptotic.

Furthermore, the comet assay methodology is utilized to check the degree of DNA cleavage caused in Caco-2 cells after exposure to compound **3e** alone and in association with Z-DEVD-FMK (a Caspase-3 inhibitor) before the compound **3e** treatment. As displayed in Table 2, there was a substantial variance in the Olive tail moment value for cells that had been treated with compound **3e** individually and those cells that had also been treated with a Caspase-3 inhibitor before compound **3e** treatment when compared to untreated cells.

**Figure 8:**
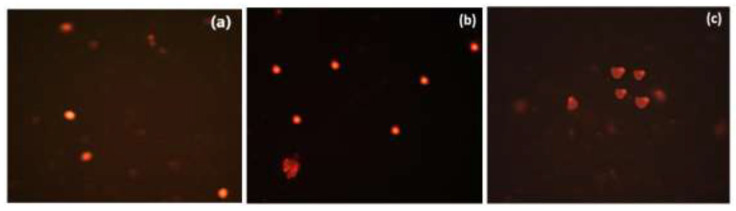
Fluorescence microscopy images of Caco-2 cells showing comet assay results: (**a**) control untreated cells; (**b**) cells treated with compound **3e** after pre-treatment with Caspase-3 inhibitor; (**c**) cells treated with compound **3e** at IC50 concentration without Caspase-3 inhibitor pre-treatment.

Moreover, fluorescence microscopy images (Figure 8) revealed that in the control untreated cells (Figure 8a) and the compound **3e**-treated cells that had been pretreated with the caspase inhibitor (Figure 8b) entire nuclei were visible, whereas a comet-like structure was seen in the compound **3e**-treated Caco-2 cells only (Figure 8c), indicating that apoptosis induction in compound **3e**-treated Caco-2 cells was dependent on Caspase-3 activation through an intrinsic mechanism.

### 2.5. Possible Apoptotic Pathways in Compound **3e**-Treated Caco-2 Cells

#### 2.5.1. Quantification Analysis Using qRT-PCR

**Figure 9:**
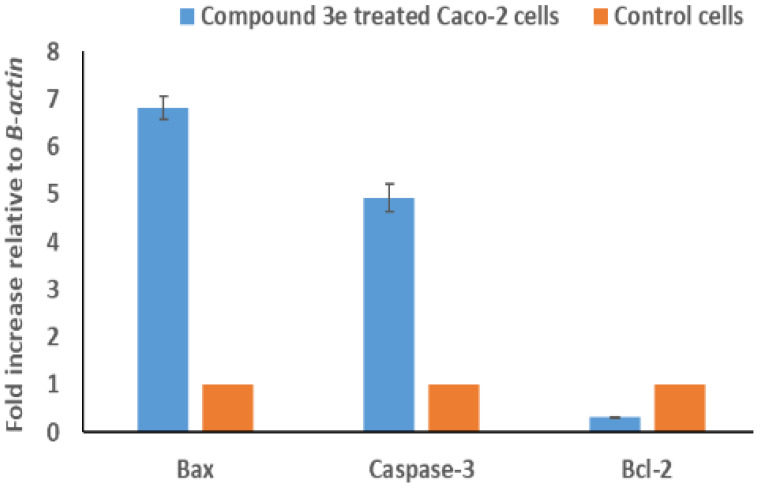
The influence of the IC_50_ concentration of compound **3e** on the *BAX*, *Caspase-3*, and *BCL-2* expression levels in Caco-2-treated cells compared to untreated cells were monitored employing qRT-PCR. Three successive assessments’ results are reported as their mean ± SD, *p* < 0.001.

The group of BCL-2 proteins, in accordance with the findings of [39], controls apoptosis by working as either pro-apoptotic regulators (BAX, BAK, and BAD) or anti-apoptotic regulators (BCL-2 and BCL-XL). Further, BAX upregulation leads to mitochondrial permeabilization, which promotes Cytochrome c release towards the cytoplasm and Caspase-3 activation [40]. So, when Caspase-3 is activated, DNA is damaged, apoptosis is carried out, and *BCL-2* gene expression is decreased [41]. In the current study, the influence of compound **3e** upon the messenger RNA (mRNA) expression of genes linked to apoptosis (*BCL2*, *BAX,* and *Caspase-3*) was assessed. The findings demonstrated that both *BAX* and *Caspase-3* levels of expression increased considerably in connection with a substantial reduction in *BCL-2* (Figure 9). These findings suggest that the intrinsic mitochondrial route may have contributed to the induction of apoptosis in the compound **3e** treated Caco-2 cells.

#### 2.5.2. Quantitative Examination of the Proteins Connected to Apoptosis Using the ELISA Testing

**Figure 10:**
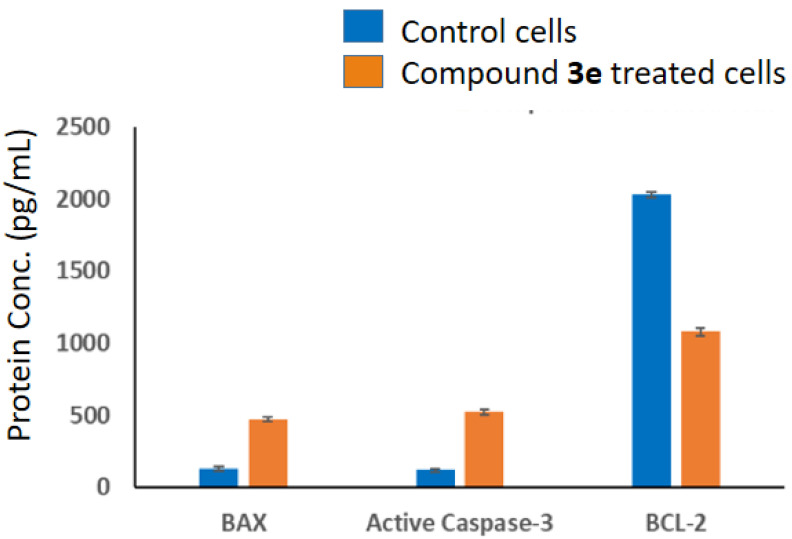
The IC_50_ dose of compound **3e**’s impact on the protein content of BAX, BCL-2, and cleaved Caspase-3 in Caco-2-treated cells as compared to control cells was investigated utilizing an ELISA method. Three different experiments’ average standard deviations are used to represent the findings; *p* < 0.001.

The proteins generated using the three studied genes, *BAX*, *Caspase-3*, and *BCL-2*, were checked out utilizing an ELISA assay in the **3e**-treated Caco-2 cells. The outcome of the experiment is shown in Figure 10, which confirms that treated cells exhibit increased levels of BAX, cleaved Caspase-3, and decreased BCL-2 proteins versus untreated ones. These findings were consistent with those of the abovementioned qRT-PCR which showed that the investigated **3e** compound considerably increased variation in apoptosis-related proteins in Caco-2-treated cells compared to control cells.

## 3. Experimental

### 3.1. Instruments and Apparatus

All reagents and solvents were purchased from Merck (Darmstadt, Germany) and used exactly as received; commercially available solvents were used for crystallizations of the obtained products without further purification. Thin-layer chromatography (TLC) on pre-coated silica gel F254 aluminum sheets from Merck was used to monitor reaction progress and purity, and compounds were visualized by exposing them to a UV lamp (model BVL-6). The melting points were measured using a Fisher-Johns apparatus (Model 12-144) and are uncorrected. The NMR spectra were carried out at ambient temperature (~25 °C) on a Brucker, Ascend, Aeon (Billerica, MA, USA) 400 MHz spectrophotometer, NMR Unit, Faculty of Pharmacy, Mansoura University, Mansoura, Egypt. Elemental analyses were conducted at the Regional Centre for Mycology and Biotechnology at Al-Azhar University in Cairo, Egypt.

### 3.2. General Method for Synthesis of Chalcones **3a**–**k**

To a mixture of heteroaromatic ketone (1.2 mmol, 1.0 equiv.) in 20 mL ethanol, and NaOH (1.4 mmol, 1.2 equiv.) in 5 mL H_2_O at 0 °C, cinnamaldehyde or its derivatives (1.2 mmol, 1.0 equiv.) were gradually added. The mixture was allowed to warm to room temperature and was stirred for 3 h, after which the precipitate of the product was collected using suction filtration on a Buchner funnel and washed repeatedly with cold water. The residue was redissolved in DCM and extracted, washed with H_2_O (1 × 40 mL), dried over MgSO_4_, filtered, evaporated to dryness, and recrystallized from ethanol.

#### 3.2.1. (2*E*,4*E*)-5-phenyl-1-(pyridin-2-yl)penta-2,4-dien-1-one **3a**

Pale yellow crystal, 89% yield; m.p. 182 °C [42]. ^1^H NMR (400 MHz, DMSO-d6): δ 8.75 (d, *J* = 4.0 Hz, 1H), 8.06 (d, *J* = 7.6 Hz, 1H), 8.00 (t, *J* = 7.5 Hz, 1H), 7.81 (d, *J* = 15.3 Hz, 1H), 7.64 (d, *J* = 9.5 Hz, 2H), 7.60 (d, *J* = 7.2 Hz, 2H), 7.38 (t, *J* = 7.2 Hz, 2H), 7.31 (dd, *J* = 16.6, 9.2 Hz, 2H) and 7.20 (d, *J* = 15.6 Hz, 1H) ppm. ^13^C NMR (101 MHz, DMSO-d6): δ 188.66 (CO), 153.51 (C), 149.12 (CH), 144.62 (CH), 142.32 (CH), 137.68 (CH), 136.04 (C), 129.31 (CH), 128.91 (CH), 127.59 (CH), 127.50 (CH), 127.47 (CH), 124.44 (CH) and 122.36 (CH) ppm. C_16_H_13_NO requires C, 81.66; H, 5.58; N, 5.95%, found: C, 81.54; H, 5.62; N, 5.92.

#### 3.2.2. (2*E*,4*E*)-5-phenyl-1-(pyridin-3-yl)penta-2,4-dien-1-one **3b**

Yellow crystal, 79% yield; m.p. 104 °C [43]. ^1^H NMR (400 MHz, DMSO-d6): δ 9.17 (d, *J* = 1.5 Hz, 1H), 8.79 (dd, *J* = 4.7, 1.4 Hz, 1H), 8.34–8.31 (m, 1H), 7.60–7.54 (m, 4H), 7.41 (m, 3H), 7.37–7.35 (m, 1H) and 7.24 (d, *J* = 5.2 Hz, 2H) ppm. ^13^C NMR (101 MHz, DMSO-d6): δ 188.59 (CO), 153.15 (CH), 149.30 (CH), 145.25 (CH), 142.53 (CH), 136.94 (C), 135.72 (CH), 132.98 (C), 129.42 (CH), 128.98 (CH), 127.41 (CH), 127.11 (CH), 125.29 (CH) and 124.00 (CH) ppm. C_16_H_13_NO requires C, 81.66; H, 5.58; N, 5.95%, found: C, 81.70; H, 5.53; N, 5.99.

#### 3.2.3. (2*E*,4*E*)-5-phenyl-1-(thiophen-2-yl)penta-2,4-dien-1-one **3c**

Colorless crystal, 95% yield; m.p. 102 °C [44]. ^1^H NMR (400 MHz, DMSO-d6): δ 8.05 (d, *J* = 3.6 Hz, 1H), 8.01 (d, *J* = 4.8 Hz, 1H), 7.58 (d, *J* = 7.3 Hz, 2H), 7.55–7.49 (m, 1H), 7.39 (t, *J* = 7.3 Hz, 2H), 7.34 (d, *J* = 6.6 Hz, 2H), 7.28–7.26 (m, 1H) and 7.20 (d, *J* = 7.6 Hz, 2H) ppm. ^13^C NMR (101 MHz, DMSO-d6): δ 181.68 (CO), 145.47 (C), 143.71 (CH), 142.01 (CH), 136.07 (C), 135.33 (CH), 132.95 (CH), 129.39 (CH), 129.05 (CH), 127.42 (CH), 127.06 (CH) and 125.31 (CH) ppm. C_15_H_12_O_2_S requires C, 79.97; H, 5.03, found: C, 79.93; H, 5.10.

#### 3.2.4. (2*E*,4*E*)-1-(furan-2-yl)-5-phenylpenta-2,4-dien-1-one **3d**

Yellow crystal, 67% yield; m.p. 112 °C [45]. ^1^H NMR (400 MHz, DMSO-d6): δ 8.01 (s, 1H), 7.58 (d, *J* = 7.8 Hz, 2H), 7.54 (d, *J* = 3.4 Hz, 1H), 7.50 (dd, *J* = 7.6, 2.4 Hz, 1H), 7.39 (t, *J* = 7.2 Hz, 2H), 7.34 (d, *J* = 7.4 Hz, 1H), 7.20 (d, *J* = 5.5 Hz, 2H), 7.15 (s, 1H) and 6.77–6.68 (m, 1H) ppm. ^13^C NMR (101 MHz, DMSO-d6): δ 177.16 (CO), 153.22 (C), 148.34 (CH), 143.62 (CH), 142.12 (CH), 136.16 (C), 129.53 (CH), 129.17 (CH), 127.55 (CH), 127.17 (CH), 125.35 (CH), 118.97 (CH) and 113.06 (CH) ppm. C_15_H_12_O_2_ requires C, 80.34; H, 5.39, found: C, 80.29; H, 5.35.

#### 3.2.5. (2*E*,4*E*)-5-phenyl-1-(1H-pyrrol-2-yl)penta-2,4-dien-1-one **3e**

Colorless crystal, 72% yield; m.p. 170 °C [46]. ^1^H NMR (400 MHz, DMSO-d6): δ 11.84 (brs., 1H, NH), 7.47–7.41 (m, 1H), 7.37 (dd, *J* = 12.7, 5.6 Hz, 2H), 7.31 (d, *J* = 7.4 Hz, 1H), 7.23–7.03 (m, 4H) and 6.25 (d, *J* = 2.4 Hz, 1H) ppm. ^13^C NMR (101 MHz, DMSO-d6): δ 178.53 (CO), 141.75 (CH), 140.77 (CH), 136.54 (C), 133.42 (C), 129.47 (CH), 129.38 (CH), 127.66 (CH), 127.59 (CH), 126.96 (CH), 126.92 (CH), 117.44 (CH) and 110.84 (CH) ppm. C_15_H_13_NO requires C, 80.69; H, 5.87; N, 6.27%, found: C, 80.74; H, 5.82; N, 6.32.

#### 3.2.6. (2*E*,4*E*)-5-(2-methoxyphenyl)-1-(pyridin-2-yl)penta-2,4-dien-1-one **3f**

Pale yellow crystal, 85% yield; m.p. 195 °C. ^1^H NMR (400 MHz, DMSO-d6): δ 8.74 (d, *J* = 2.8 Hz, 1H), 8.05 (d, *J* = 7.5 Hz, 1H), 7.99 (t, *J* = 7.6 Hz, 1H), 7.77 (d, *J* = 15.2 Hz, 1H), 7.66–7.59 (m, 3H), 7.38 (d, *J* = 15.7 Hz, 1H), 7.35–7.25 (m, 2H), 7.02 (d, *J* = 8.3 Hz, 1H), 6.96 (t, *J* = 7.5 Hz, 1H) and 3.83 (s, 3H, OCH_3_) ppm. ^13^C NMR (101 MHz, DMSO-d6): δ 188.79 (CO), 157.35 (C), 153.65 (C), 149.15 (CH), 145.48 (CH), 137.72 (CH), 137.28 (CH), 130.89 (CH), 128.10 (CH), 127.68 (CH), 127.50 (CH), 124.45 (C), 124.00 (CH), 122.40 (CH), 120.81 (CH), 111.69 (CH) and 55.98 (CH_3_) ppm. C_17_H_15_NO_2_ requires C, 76.94; H, 5.70; N, 5.28%, found: C, 77.01; H, 5.74; N, 5.24.

#### 3.2.7. (2*E*,4*E*)-5-(2-methoxyphenyl)-1-(1H-pyrrol-2-yl)penta-2,4-dien-1-one **3g**

Pale yellow crystal, 75% yield; m.p. 177 °C. ^1^H NMR (400 MHz, DMSO-d6): δ 11.92 (brs, 1H, NH), 7.59 (d, *J* = 7.6 Hz, 1H), 7.44 (dd, *J* = 14.8, 10.4 Hz, 1H), 7.35–7.29 (m, 1H), 7.26–7.16 (m, 3H), 7.12 (d, *J* = 6.5 Hz, 2H), 7.03 (d, *J* = 8.3 Hz, 1H), 7.00–6.92 (m, 1H), 6.25 (d, *J* = 2.1 Hz, 1H) and 3.84 (s, 3H, OCH_3_) ppm. ^13^C NMR (101 MHz, DMSO-d6): δ 178.20 (CO), 157.20 (C), 148.06 (CH), 142.00 (CH), 135.29 (CH), 133.19 (C), 130.48 (CH), 128.01 (CH), 127.51 (CH), 126.25 (CH), 124.67 (C), 120.86 (CH), 116.73 (CH), 111.72 (CH), 110.29 (CH) and 56.02 (CH_3_) ppm. C_16_H_15_NO_2_ requires C, 75.87; H, 5.97; N, 5.53%, found: C, 75.84; H, 5.94; N, 5.59.

#### 3.2.8. (2*E*,4*E*)-5-(4-nitrophenyl)-1-(pyridin-2-yl)penta-2,4-dien-1-one **3h**

Yellow crystal, 84% yield; m.p. 205 °C. ^1^H NMR (400 MHz, DMSO-d6): δ 8.78 (d, *J* = 4.4 Hz, 1H), 8.24 (d, *J* = 8.5 Hz, 1H), 8.06 (dd, *J* = 16.5, 7.5 Hz, 2H), 7.92–7.86 (m, 3H), 7.73–7.66 (m, 2H), 7.67–7.52 (m, 2H) and 7.36 (d, *J* = 14.9 Hz, 1H) ppm. ^13^C NMR (101 MHz, DMSO-d6): δ 188.92 (CO), 153.34 (C), 149.29 (CH), 147.21 (C), 143.57 (CH), 142.72 (C), 139.47 (CH), 137.91 (CH), 132.02 (CH), 128.39 (CH), 127.83 (CH), 126.79 (CH), 124.20 (CH) and 122.55 (CH) ppm. C_16_H_12_N_2_O_3_ requires C, 68.59; H, 4.32; N, 9.99%, found: C, 68.63; H, 4.37; N, 9.95.

#### 3.2.9. (2*E*,4*E*)-5-(4-nitrophenyl)-1-(pyridin-3-yl)penta-2,4-dien-1-one **3i**

Yellow crystal, 74% yield; m.p. 125 °C [47]. ^1^H NMR (400 MHz, DMSO-d6): δ 9.19 (d, *J* = 1.8 Hz, 1H), 8.84 (dd, *J* = 4.8, 1.5 Hz, 1H), 8.36 (dt, *J* = 8.0, 1.8 Hz, 1H), 8.28 (d, *J* = 8.7 Hz, 2H), 7.63 (dd, *J* = 8.3, 4.7 Hz, 2H), 7.55 (d, *J* = 16.7 Hz, 2H), 7.52–7.44 (m, 2H) and 7.40 (d, *J* = 15.4 Hz, 1H) ppm. ^13^C NMR (101 MHz, DMSO-d6): δ 189.14 (CO), 153.80 (CH), 149.83 (CH), 148.23 (C), 144.47 (CH), 142.94 (C), 139.96 (CH), 136.26 (CH), 133.23 (C), 131.84 (CH), 128.71 (CH), 128.04 (CH), 124.63 (CH), and 124.51 (CH) ppm. C_16_H_12_N_2_O_3_ requires C, 68.59; H, 4.32; N, 9.99%, found: C, 68.55; H, 4.35; N, 9.93.

#### 3.2.10. (2*E*,4*E*)-5-(4-nitrophenyl)-1-(thiophen-2-yl)penta-2,4-dien-1-one **3j** [48]

Pale yellow, 91% yield; m.p. 218 °C (lit. 210). ^1^H NMR (400 MHz, DMSO-d6): δ 8.07–8.03 (m, 3H), 7.84 (d, *J* = 8.4 Hz, 2H), 7.72 (d, *J* = 8.4 Hz, 1H), 7.56–7.46 (m, 3H) and 7.32–7.30 (m, 2H) ppm. ^13^C NMR (101 MHz, DMSO-d6): δ 181.50 (CO), 147.14 (C), 145.20 (C), 142.60 (C), 142.46 (CH), 138.99 (CH), 135.78 (CH), 133.26 (CH), 131.34 (CH), 129.09 (CH), 128.20 (CH), 127.60 (CH), and 124.21 (CH) ppm. C_15_H_11_NO_3_S requires C, 63.14; H, 3.89; N, 4.91%, found: C, 63.20; H, 3.84; N, 4.95.

#### 3.2.11. (2*E*,4*E*)-1-(furan-2-yl)-5-(4-nitrophenyl)penta-2,4-dien-1-one **3k** [49,50]

Yellow, 65% yield; m.p. 189 °C (lit 188.5–189.5 °C). ^1^H NMR (400 MHz, DMSO-d6): δ 8.26–8.21 (m, 1H), 8.05 (d, *J* = 10.0 Hz, 1H), 7.84 (d, *J* = 8.7 Hz, 1H), 7.58–7.52 (m, 1H), 7.44–7.40 (m, 1H), 7.35–7.25 (m, 1H) and 6.77 (d, *J* = 13.7 Hz, 1H) ppm. ^13^C NMR (101 MHz, DMSO-d6): δ 176.74 (CO), 152.93 (C), 148.48 (CH), 147.14 (C), 142.62 (C), 142.32 (CH), 139.04 (CH), 131.37 (CH), 128.22 (CH), 127.53 (CH), 124.21 (CH), 119.16 (CH) and 112.99 (CH) ppm. C_15_H_11_NO_4_ requires C, 66.91; H, 4.12; N, 5.20%, found: C, 66.95; H, 4.17; N, 5.15.

### 3.3. Antioxidant Activity for the Tested Compounds

The potential of several compounds to scavenge DPPH free radicals was quantified employing a colorimetric approach [51]. Various doses of all tested compounds (**3a**–**3k**) were prepared in methanol (40–200 µM), and standard solutions of ascorbic acid (40–200 µM) were made by dissolving the compound in methanol. After determining the optimal concentration of each compound and ascorbic acid, the solutions were supplemented with DPPH. The radical scavenging capacity (RSC) was calculated by measuring the absorbance at 517 nm and using the Formula (1):

% DPPH RSC = (Abs. of control − Abs. of sample or standard)/Abs. of control × 100(1)

### 3.4. Cytotoxicity Analysis

#### 3.4.1. Development of the Tested Cell Lines

The American Type Culture Collection (ATCC) provided WI-38 (ATCC CCL-75), a cell line from the healthy human embryonic lung, and Caco-2 (ATCC HTB-37), a cell line from colon cancer. These cells were developed in DMEM medium that was supplemented with antibiotics and fetal bovine serum in a 5% CO_2_ humidified incubator at 37 °C.

#### 3.4.2. Cytotoxicity Analysis Using MTT Experiment

The toxicity of the tested compounds (**3a**–**3k**) toward Caco-2 cancer cells was examined and compared to 5-Fluorouracil (5-FU, the standard reference drug) utilizing MTT assay, as previously explained [51,52]**.** In brief, 1 × 10^5^ Caco-2 cells/mL were distributed into each well of a 96-well plate and kept overnight at 37 °C in their growth mixture. The cells were then subjected to the various doses of the studied compounds (50–1000 µM). Following that, the cells were exposed to MTT solution, which induced formazan formation from the MTT, a byproduct of the metabolism of MTT that is proportionate to the quantity of intact cells. To further dissolve the formazan crystal, dimethyl sulfoxide was applied to the cells. Caco-2 survival was obtained through measuring the value of the optical density at 570 nm implementing a microplate reader (Bio-Rad, Hercules, CA, USA), and the effect of the substances on cell proliferation was expressed using the aforementioned formula (2):

Cell viability % = (Abs. of tested sample − Abs. of blank)/(Abs. of control − Abs. of blank) × 100(2)

The half-suppression (IC_50_) dose of the test drugs over a 24 h incubation period was identified through examination of the concentration–response curve. Each value was evaluated three times, and the average of the three ratings was employed to obtain the results.

The effects of the investigated compounds on the survival of healthy cells, WI38, have been evaluated and compared to 5-Fluorouracil (5-FU, the standard reference drug) utilizing the MTT test at concentrations ranging from 50 to 1000 µM to further determine whether the substances were secure for usage on living tissues. Three distinct evaluations of each value were carried out to calculate the final findings.

#### 3.4.3. The ELISA Test for Determining Lactate Dehydrogenase Levels

The amount of lactate dehydrogenase enzyme (LDH) secreted by compound **3e**-treated Caco-2 cells was measured using the double-antibody-sandwich ELISA method (Sun-Red, Cat. N. 201-28-0094) to confirm the cytotoxic impact of the tested chemical on Caco-2 cells. Specifically, 40 µL of the supernatant obtained from centrifuging the Caco-2 cells that had been treated with the **3e** compound was added to the test wells. Next, LDH-antibody (10 µL) and Streptavidin-HRP (50 µL) were transferred to every well. After 60 min of incubation at 37 °C, the 96-well plate was shaken gently. LDH-antibody (10 µL) and Streptavidin-HRP (50 µL) were then added to each well for analysis. The 96-well plate was then gently shaken and left to incubate for 60 min at 37 °C. The plates were then washed, and each test well received 50 µL of chromogen solution A and 50 µL of chromogen solution B. The plates were then incubated at 37 °C, and darkened for 10 min. Stop solution (50 µL) was then applied to each well. Once testing was complete, 450 nm was used to determine the plate’s optical density (OD). The amount of released LDH was calculated using the standard curve that had been previously established.

### 3.5. Apoptosis Analyses

#### 3.5.1. Evaluation of Apoptosis via Annexin V/PI Investigation

The annexin V/PI assay focuses on the translocation of phosphatidylserine (PS) from the inner plasma membrane toward the cell surface within apoptotic cells. Accordingly, the Annexin V-FITC detection kit I (BD Biosciences) was implemented to figure out the numbers of apoptotic and necrotic cells in both the compound **3e**-treated and untreated Caco-2 cells for 24 h. [52,53]**.** In brief, Caco-2 cells were collected, expanded in 6-well culture dishes with 1 × 10^6^ cells per well, and then allowed to incubate for an overnight period to promote cell adhesion and proliferation. After only 24 h in the presence of the **3e** compound, the cells were trypsinized, centrifuged, rinsed with PBS, and then mixed with a solution containing Annexin V binding buffer (1×) before being exposed to the Annexin V-FITC and propidium iodide stains. At last, a flow cytometer (BD FACSCalibur™, Biosciences, San Jose, CA, USA) was implemented to find the amount of early as well as late apoptotic cells, and the outcomes were presented graphically.

#### 3.5.2. DNA Fragmentation Assessment

The production of genotoxicity in Caco-2 cells treated with compound **3e** was investigated using a comet test. Caco-2 cells were pretreated with a caspase-3 inhibitor (Z-DEVD-FMK) to ascertain whether or not caspase-3 activity contributed to the DNA damage induced by compound **3e**. Using the comet test [54,55,56], in order to assess the influence of compound **3e** upon DNA damage, Caco-2 cells were subjected to either compound **3e** separately (IC_50_ concentration) or compound **3e** (IC_50_ concentration) following prior treatment with Z-DEVD-FMK (50 M) for 1 h. Broken strands of broken DNA will detach from intact strands of cellular DNA during fragmentation, producing a structure similar to a comet tail visible under a fluorescence microscope (Carl Zeiss, Axiostar Plus 1169–149, Jena, Germany). For each slide, 100 photos of comets of varying shapes were captured using a computerized image analysis system. Afterwards, the photos were analyzed using the TriTek Comet ScoreTM program (TriTek Corp., Sumerduck, VA, USA) to gather information about the comet’s properties. The tail DNA and tail moment, two often-utilized markers, were built to be used in discovering the data. The Olive tail moment (OTM), which is also called a tail moment parameter, is generally accepted as the gold standard for evaluating DNA damage. Its value, which was calculated using Equation (3), is proportional to the tail’s DNA mobility and DNA abundance.

OTM = quantity of tail moment × quantity of tail DNA divided by 100(3)

### 3.6. Potential Apoptosis Mechanisms

#### 3.6.1. Quantitative Real-Time PCR Technique (qRT-PCR)

The relative amounts of messenger RNA (mRNA) for apoptosis-related genes (*BCL-2*, *Caspase-3*, and *BAX*) have been monitored in order to look into potential processes relating to the effect of the tested **3e** compound on apoptosis inside the treated Caco-2 cancer cells. In a nutshell, Thermo Fisher Scientific’s RNA purification kit (Waltham, MA, USA) (catalog #K0731) was used to isolate total RNA from both control- and Caco-2-treated cells, as per the manufacturer’s instructions. The ratio of A260/A280 was then quantified using a spectrophotometer to figure out the amounts of extracted RNA in each sample. Then, following the manufacturer’s instructions, first-strand cDNA was produced from the collected RNA samples using a kit made by Thermo Fisher Scientific called RevertAid (catalog #K1621). Real-time PCR amplification was performed on an Applied Biosystems StepOnePlusTM instrument using the Thermo Fisher Scientific Maxima SYBR Green qPCR kit (catalog #K0221) and gene-specific primers (Invitrogen, Waltham, MA, USA), as detailed in Table 3. Then, the relative quantification (RQ) of the utilized genes was calculated [57] using a comparative threshold cycle approach, compared against their varied expression in the untreated samples, and normalized to B-actin as a housekeeping gene. These findings were averaged over three separate investigations, with triplicates of each experiment being conducted.

#### 3.6.2. ELISA Analysis

Protein expression levels of the under investigation genes were evaluated in Caco-2 cells treated with compound **3e** using an ELISA assay. Three different ELISA kits were used: one for BAX (ab199080), one for cleaved Caspase-3 (ab220655), and one for BCL-2 (ab272102), which were supplied via Abcam (Cambridge, UK). All of these kits are based on a single, highly sensitive sandwich enzyme assay that lasts 90 min and uses a precoated ELISA plate and an anti-tag antibody to capture antibodies that have been labeled with an affinity tag. The tested compound **3e**-treated cultured Caco-2 cells were centrifuged, the resulting pellets were discarded, and the residual supernatant was mixed with an antibody cocktail solution in a 96-well ELISA plate. The plate was then incubated for 1 h at room temperature. After thoroughly rinsing the plate in wash buffer (1×), in each well, a ten min incubation period of tetramethylbenzidine substrate solution was conducted. Following that, the plate was stored in a dim area. The intensity at 450 nm was then measured with a microplate reader immediately after a stop solution was added.

### 3.7. Statistical Analysis

Three replicates of each assay were performed, and the mean ± SD is presented. The SPSS 17.0 program was utilized for all statistical testing. Statistics were deemed significant at *p*-values ≤ 0.05, 0.01, and 0.001.

## 4. Conclusions

A number of cinnamaldehyde-based chalcones have been prepared, characterized, and evaluated for their antioxidant and inhibitory effects on human Caco-2 cancer cells. When compared to the other compounds, **3e** had the best IC_50_ value in the DPPH assay for activating antioxidant defenses and the MTT test for reducing the growth of human Caco-2 cells. Furthermore, the compound **3e**’s safety for use in live tissues has been validated through in vitro tests. Caco-2 cell-growth inhibition was studied, and its underlying mechanisms were examined, employing apoptosis detection methods (Annexin V/PI staining and comet assays), qRT-PCR, and ELISA assays. The outcomes revealed that compound **3e** inhibited the proliferation of Caco-2 cancer cells, possibly due to the activation of Caspase-3 via an intrinsic apoptotic pathway. Based on these findings, it appears that compound **3e** may be useful in the therapeutic management of human colon cancer.

## Figures and Tables

**Table 1:** The compounds’ IC_50_ values against Caco-2 cells compared to the IC_50_ value of the 5-Fluorouracil reference drug as determined using the MTT method. As the mean ± SD for the values, the findings of three different analyses are shown.

The Tested Compounds	3a	3b	3c	3d	3e	3f	3g	3h	3i	3j	3k	5-FU
**IC_50_ Conc. (µM)**	725.57 ± 3.69	1624.2 ± 4.69	1213.51 ± 4.98	1209.91 ± 4.22	32.19 ± 3.92	172.74 ± 3.84	336.17 ± 4.96	548.58 ± 3.76	206.64 ± 2.31	149.37 ± 2.55	130.38 ± 3.28	33.12 ± 1.45

**Table 2:** The variations in DNA damage between compound **3e**-treated Caco-2 cells, both with and without a prior treatment with a Caspase-3 inhibitor, and untreated Caco-2 cells were monitored, employing comet measurement variables. Three different tests’ results are shown as mean ± SD. *** *p* < 0.001.

The Studied Cells	% DNA in Tail	Tail Moment	Olive Tail Moment (OTM)
The control, untreated, Caco-2 cells	2.32 ± 0.85	0.32 ± 0.01	0.46 ± 0.03
The cultured Caco-2 cells with compound **3e** with pretreatment of Caspase-3 inhibitor	4.55± 1.73	0.44± 0.08	0.69± 0.16
The cultured Caco-2 cells with compound **3e** alone (without pretreatment of Caspase-3 inhibitor)	8.18 ± 0.56 ***	1.19 ± 0.03 ***	1.92 ± 0.04 ***

**Table 3:** Primers utilized in quantitative real-time polymerase chain reaction for the investigated genes.

Gene	Forward Primer	Reverse Primer
*BCL-2*	5′-CATGTGTGTGGAGAGCGTCAA-3′	5′-GCCGGTTCAGGTACTCAGTCA-3′
*BAX*	5′-GATCCAGGATCGAGCAGA-3′	5′-AAGTAGAAGAGGGCAACCAC-3′
*Caspase-3*	5′-CAGAACTGGACTGTGGCATTGAG-3′	5′-GGATGAACCAGGAGCCATCCT-3′
*B-Actin*	5′-AGTTGCGTTACACCCTTTCTTC-3′	5′-TCACCTTCACCGTTCCAGTTT-3′

## Data Availability

The datasets used and analyzed during the current study are available from the corresponding author upon reasonable request.

## Supplementary Materials

The following supporting information can be downloaded at: https://www.mdpi.com/article/10.3390/biom14020216/s1, File S1:Proton and carbon-13 NMR spectra for compounds **3h**–**k**.
