# Correction: El-Atawy et al. Synthesis, Characterization, Antioxidant, and Anticancer Activity Against Colon Cancer Cells of Some Cinnamaldehyde-Based Chalcone Derivatives. *Biomolecules* 2024, *14*, 216

**DOI:** 10.3390/biom16030438

**Published:** 2026-03-13

**Authors:** Mohamed A. El-Atawy, Demiana H. Hanna, Ali H. Bashal, Hoda A. Ahmed, Eida M. Alshammari, Ezzat A. Hamed, Abdullah R. Aljohani, Alaa Z. Omar

**Affiliations:** 1 Department of Chemistry, College of Science in Yanbu, Taibah University, Yanbu Governorate 30799, Saudi Arabia; maatawy@taibahu.edu.sa (M.A.E.-A.); abishil@taibahu.edu.sa (A.H.B.); hmahmoud@taibahu.edu.sa (H.A.A.); abdullah.rajaallah@gmail.com (A.R.A.); 2 Chemistry Department, Faculty of Science, Alexandria University, P.O. Box 426 Ibrahemia, Alexandria 21321, Egypt; ezzat.awad@alexu.edu.eg (E.A.H.); alaazaki@alexu.edu.eg (A.Z.O.); 3 Department of Chemistry, Faculty of Science, Cairo University, Giza 12613, Egypt; 4 Department of Chemistry, College of Sciences, University of Ha’il, Ha’il 55473, Saudi Arabia; eida.alshammari@uoh.edu.sa; 5 Saudi Irrigation Organization (SIO), Al-Hassa 31982, Saudi Arabia

In the original article [1], concerns were raised regarding Figures 7b and 8. Following a thorough investigation, the authors provide the following clarifications and corrections.

Figure 7b: Some data points appear outside the plotted axes. This phenomenon is caused by fluorescence intensities that exceed the display scale whilst remaining within the detector’s measurement range, and by particularly strong or saturated signals (for example, from double-staining) that map to axis values beyond the displayed grid. These off-grid events reflect valid detector values rather than erroneous measurements. 

Figure 8b: The apparent similarity between images in Figure 8b is explained by the biological uniformity of the samples and fully standardised imaging conditions. All cells were processed under identical conditions (same incubation, lysis, staining buffer, and timing), stained with a DNA-binding fluorescent dye that yields uniform nuclear staining, and imaged with constant microscope settings. Under these controlled conditions, intact nuclei can appear highly similar across fields. 

Figure 8c: The similarities observed among nuclei in the treated group (Figure 8c) are attributable to a uniform biological response to the DNA-damaging agent (compound **3e**). All treated samples were collected at the same time point and processed with identical electrophoresis, lysis, pH, staining, and imaging parameters, which produces comparable comet structures and fragmentation patterns across replicate samples.

To address presentation concerns and to provide the complete primary data, Figure 8 in the original article has been replaced with the uncropped original Figure 8 (Figure 8a–c). No other changes to figures or data were required. The corrected Figure 8 appears below.

**Figure 8:**
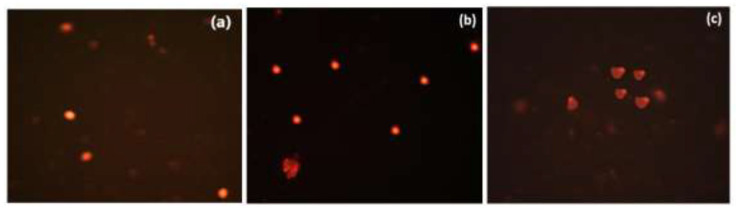
Fluorescence microscopy images of Caco-2 cells showing comet assay results: (**a**) control untreated cells; (**b**) cells treated with compound **3e** after pre-treatment with Caspase-3 inhibitor; (**c**) cells treated with compound **3e** at IC50 concentration without Caspase-3 inhibitor pre-treatment.

In the original publication, there were errors regarding the affiliations for Hoda A. Ahmed. At the request of the authors, affiliation 3 (Department of Chemistry, Faculty of Science, Cairo University, Giza 12613, Egypt) has been removed from the author's entry. Additionally, affiliation 1, which was previously listed as “Chemistry Department, Faculty of Science at Yanbu, Taibah University, Yanbu 46423, Saudi Arabia”, has been corrected to read: “Department of Chemistry, College of Science in Yanbu, Taibah University, Yanbu Governorate 30799, Saudi Arabia”.

The authors apologize for any inconvenience caused and state that the scientific conclusions are unaffected. This correction was approved by the Academic Editor. The original publication has also been updated.
