# Supplementary material for: Synthesis, Characterization, Antioxidant, and Anticancer Activity against Colon Cancer Cells of Some Cinnamaldehyde-Based Chalcone Derivatives
Source: Biomolecules. 2024 Feb 12;14(2):216. doi: 10.3390/biom14020216 (PMC10886690; doi:10.3390/biom14020216)
Supplement: Supplementary file 1 [file biomolecules-14-00216-s001.zip › biomolecules-2853232-supplementary.pdf]

Synthesis, characterization, antioxidant and anticancer activity against colon cancer cells of some cinnamaldehyde-based chalcone derivatives

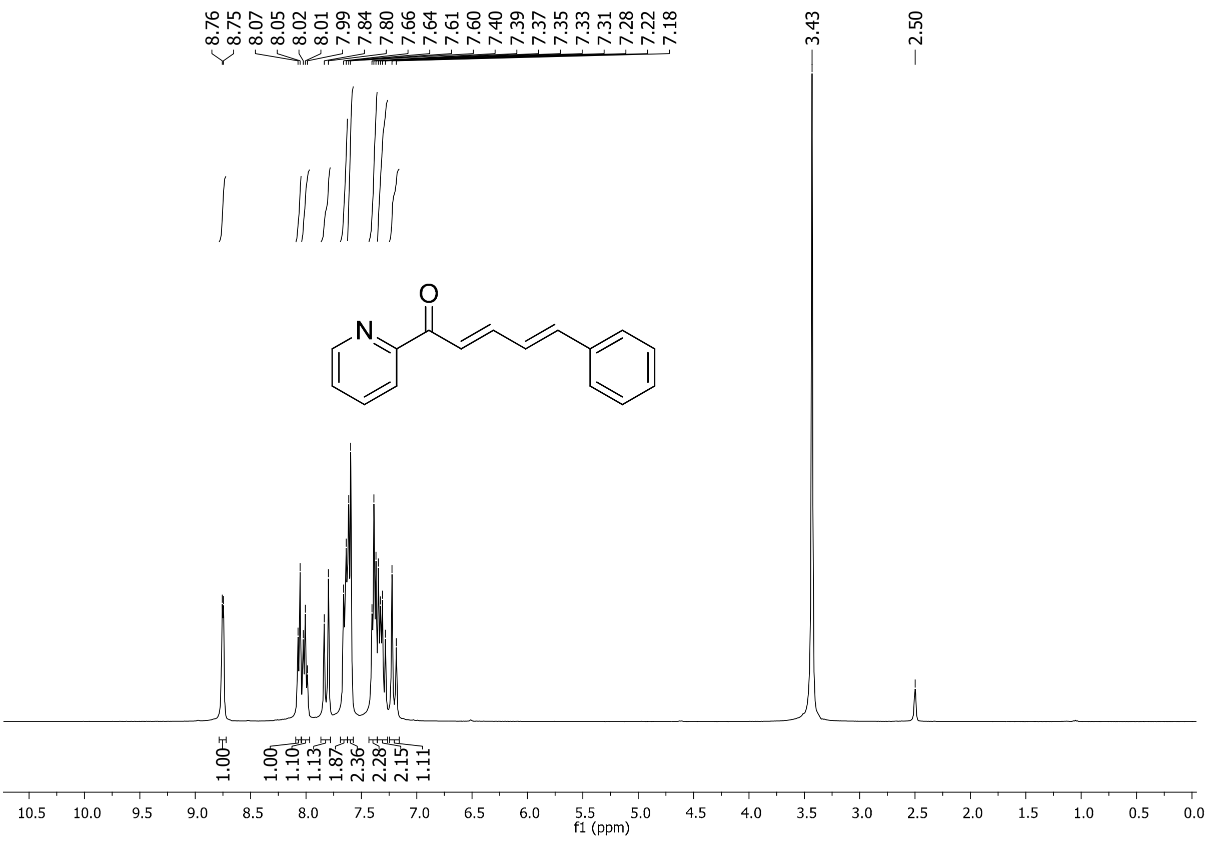

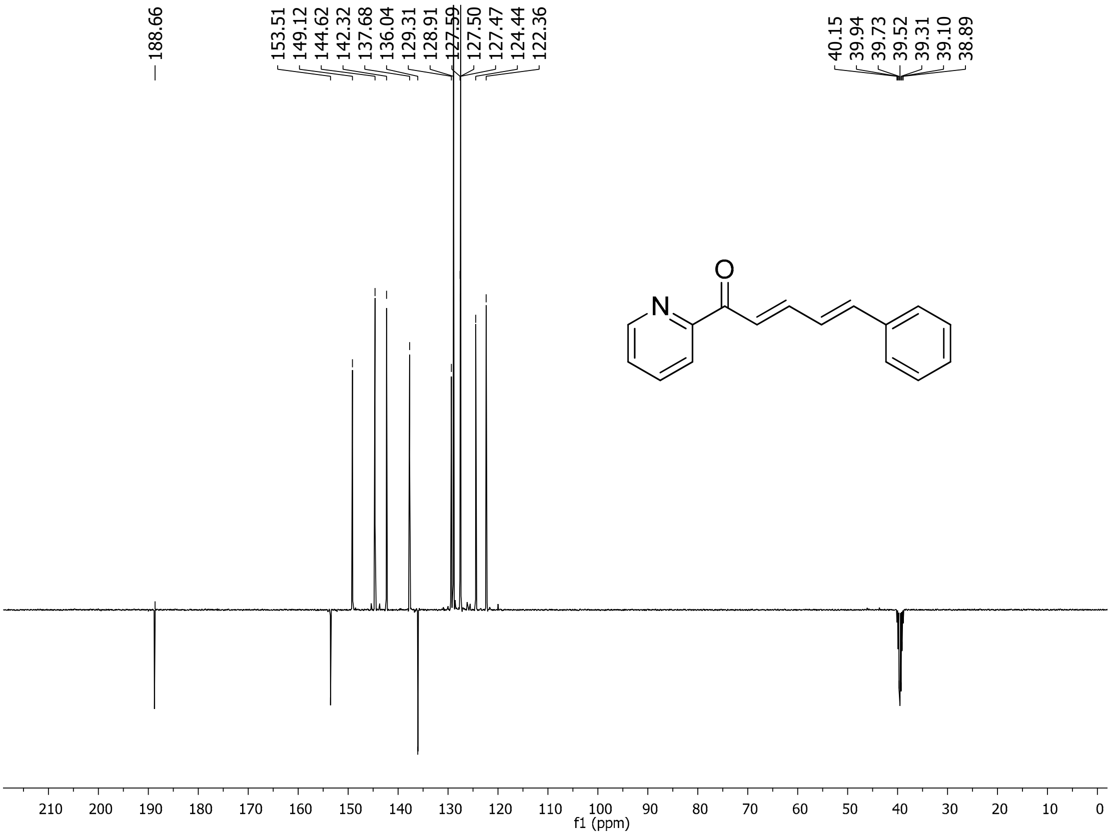

188.66
153.51
149.12
144.62
142.32
137.68
136.04
129.31
128.91
127.59
127.50
127.47
124.44
122.36
40.15
39.94
39.73
39.52
39.31
39.10
38.89
O
N
210
200
190
180
170
160
150
140
130
120
110
100
90
80
70
60
50
40
30
20
10
0
f1 (ppm)

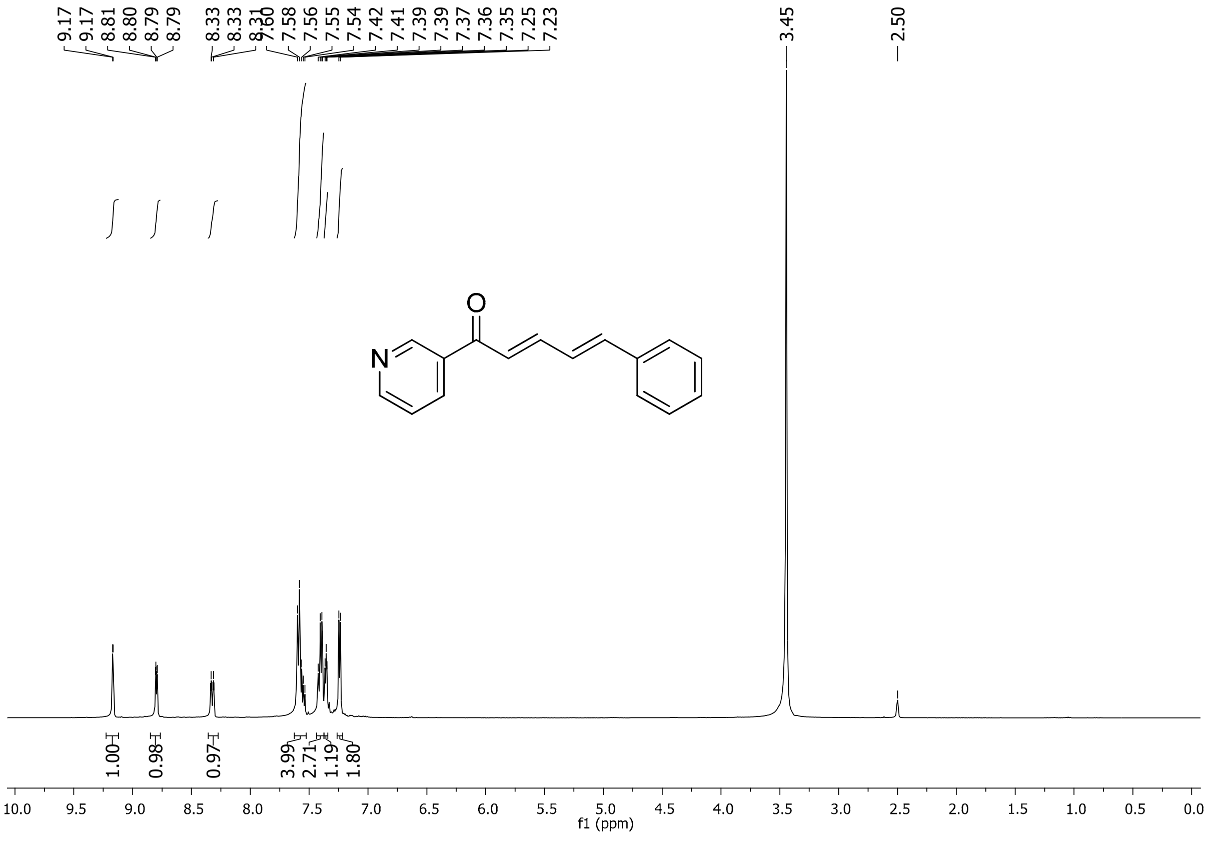

9.17
9.17
8.81
8.80
8.79
8.79
8.33
8.33
8.31
7.60
7.58
7.56
7.55
7.54
7.42
7.41
7.39
7.39
7.37
7.36
7.35
7.25
7.23
3.45
2.50
N
O
1.00
0.98
0.97
3.99
2.71
1.19
1.80
10.0
9.5
9.0
8.5
8.0
7.5
7.0
6.5
6.0
5.5
5.0
4.5
4.0
3.5
3.0
2.5
2.0
1.5
1.0
0.5
0.0
f1 (ppm)

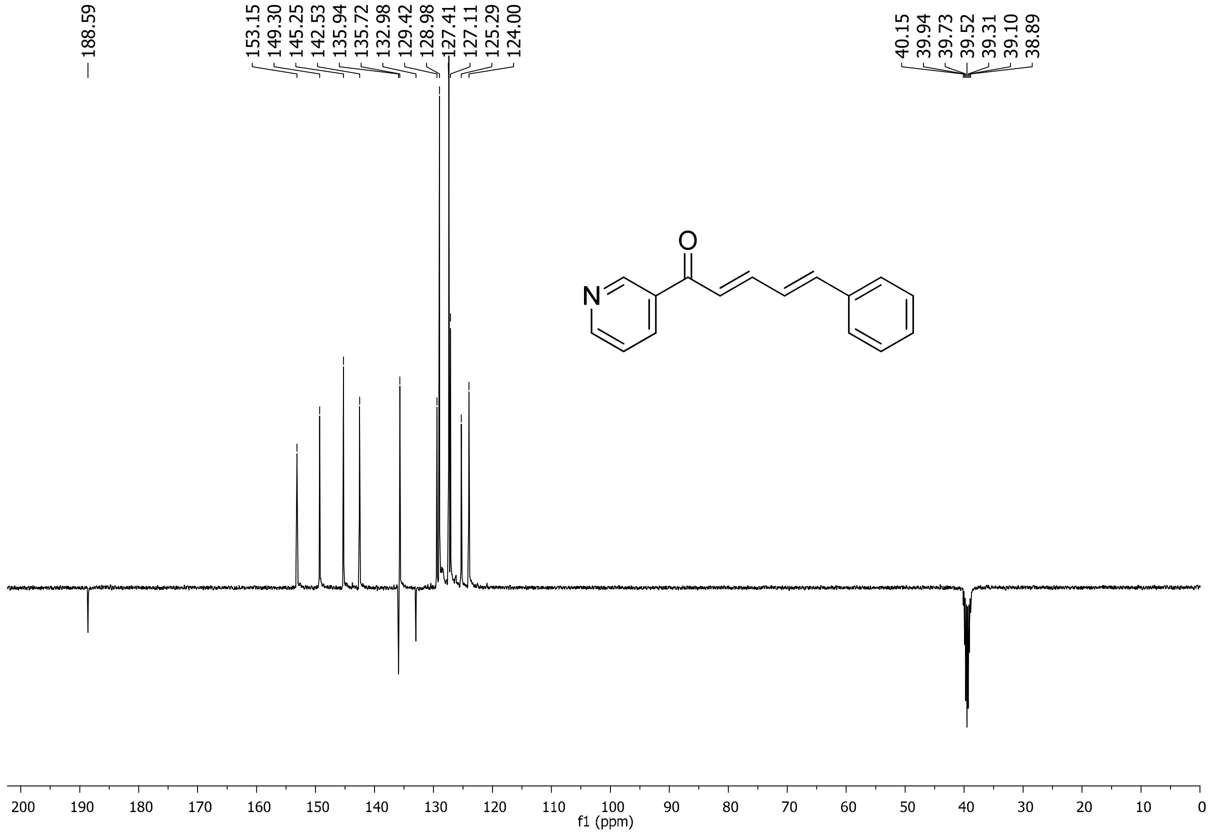

188.59
153.15
149.30
145.25
142.53
135.94
135.72
132.98
129.42
128.98
127.41
127.11
125.29
124.00
40.15
39.94
39.73
39.52
39.31
39.10
38.89
O
N
200 190 180 170 160 150 140 130 120 110 100 90 80 70 60 50 40 30 20 10 0
f1 (ppm)

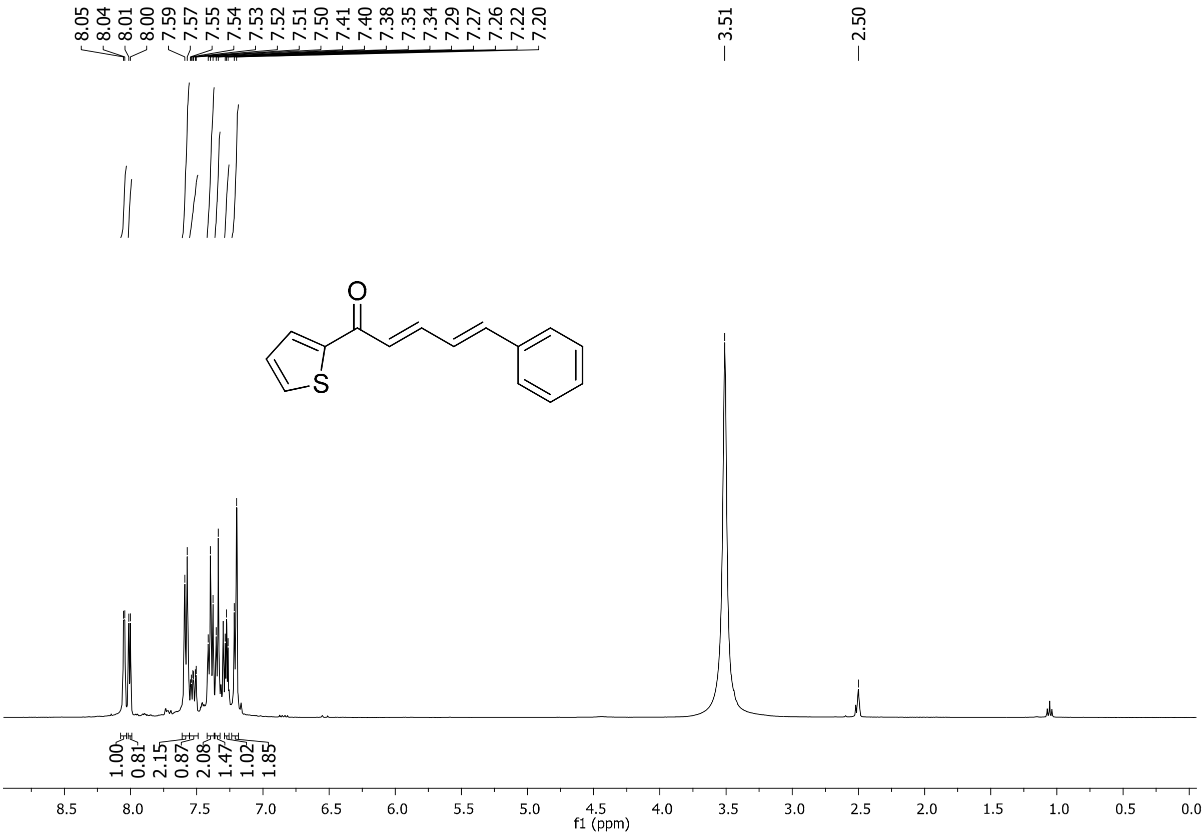
8.05
8.04
8.01
8.00
7.59
7.57
7.55
7.54
7.53
7.52
7.51
7.50
7.41
7.40
7.38
7.35
7.34
7.29
7.27
7.26
7.22
7.20
3.51
2.50
O
S
1.00
0.81
2.15
0.87
2.08
1.47
1.02
1.85
8.5
8.0
7.5
7.0
6.5
6.0
5.5
5.0
4.5
4.0
3.5
3.0
2.5
2.0
1.5
1.0
0.5
0.0
f1 (ppm)

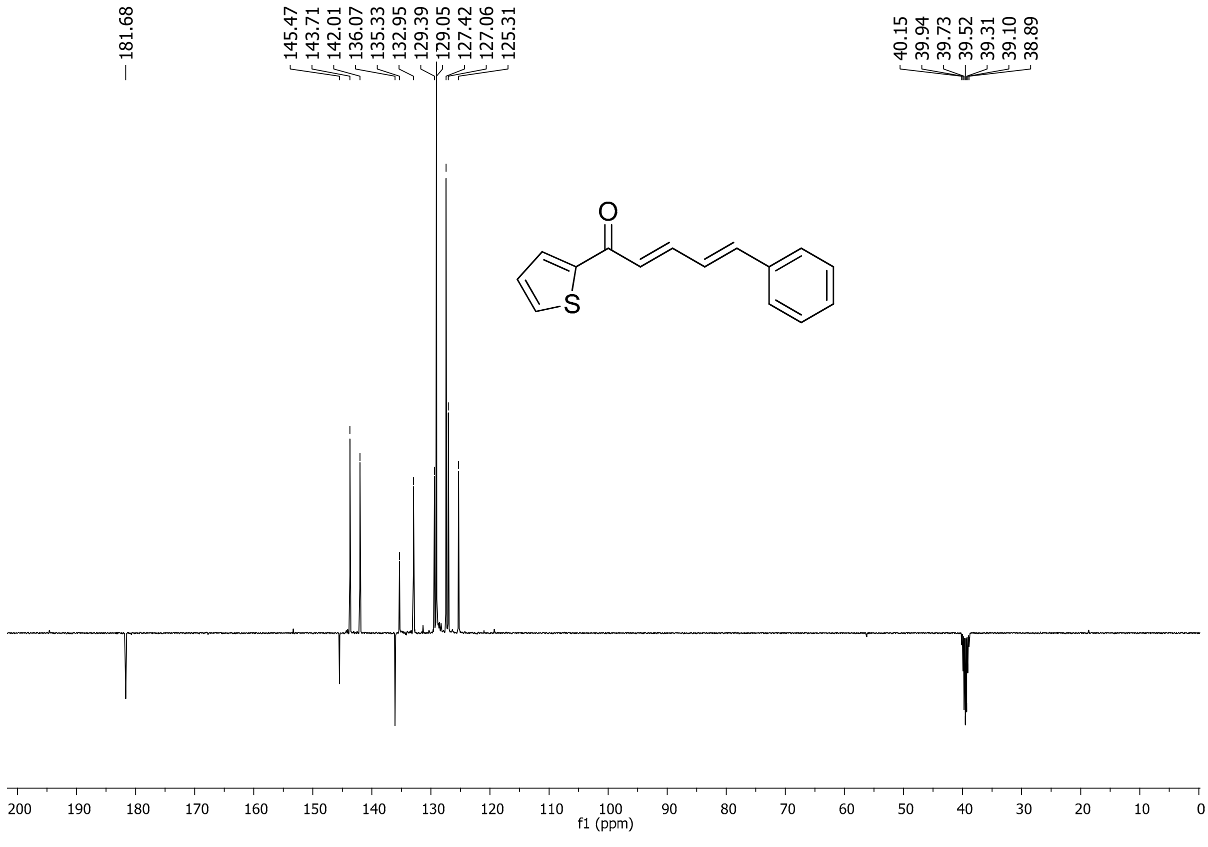

181.68
145.47
143.71
142.01
136.07
135.33
132.95
129.39
129.05
127.42
127.06
125.31
40.15
39.94
39.73
39.52
39.31
39.10
38.89
O
S
200 190 180 170 160 150 140 130 120 110 100 90 80 70 60 50 40 30 20 10 0
f1 (ppm)

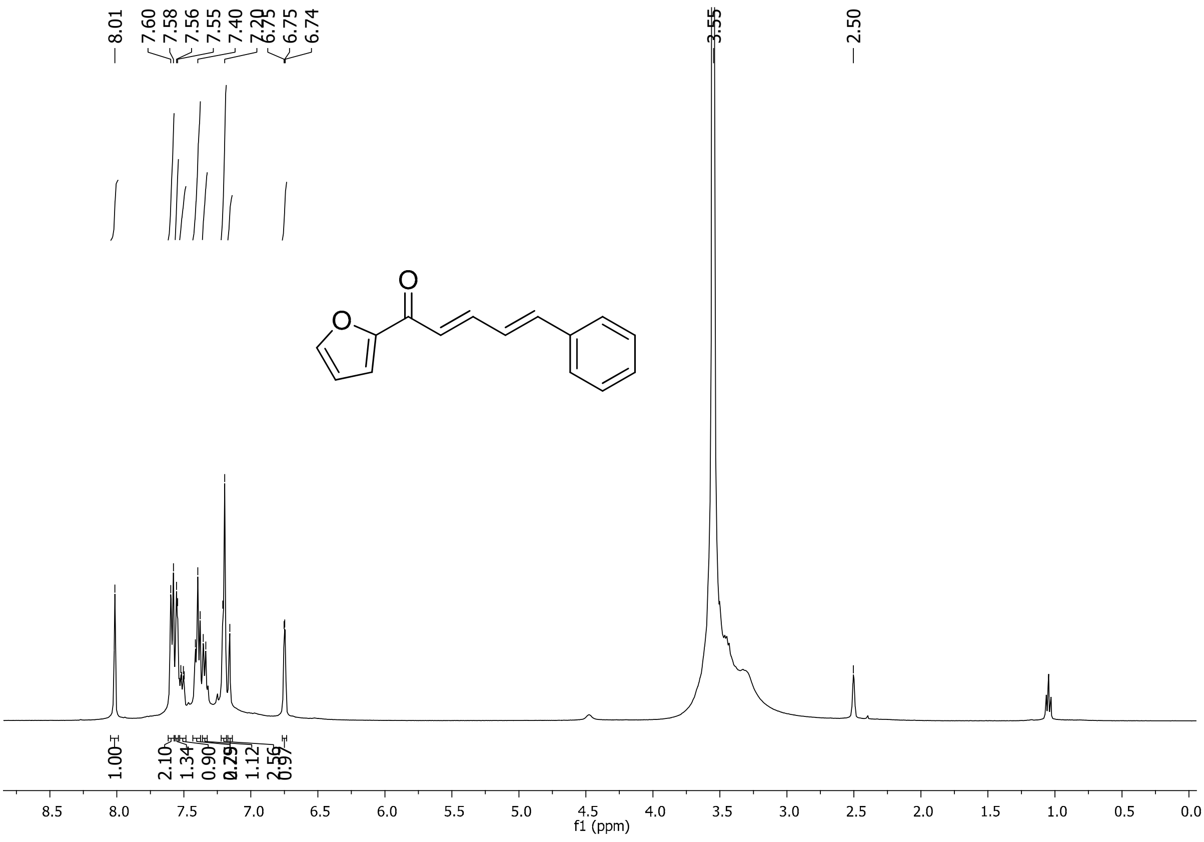
8.01
7.60
7.58
7.56
7.55
7.40
7.20
6.75
6.75
6.74
3.55
2.50
1.00
2.10
1.34
0.90
1.12
f1 (ppm)

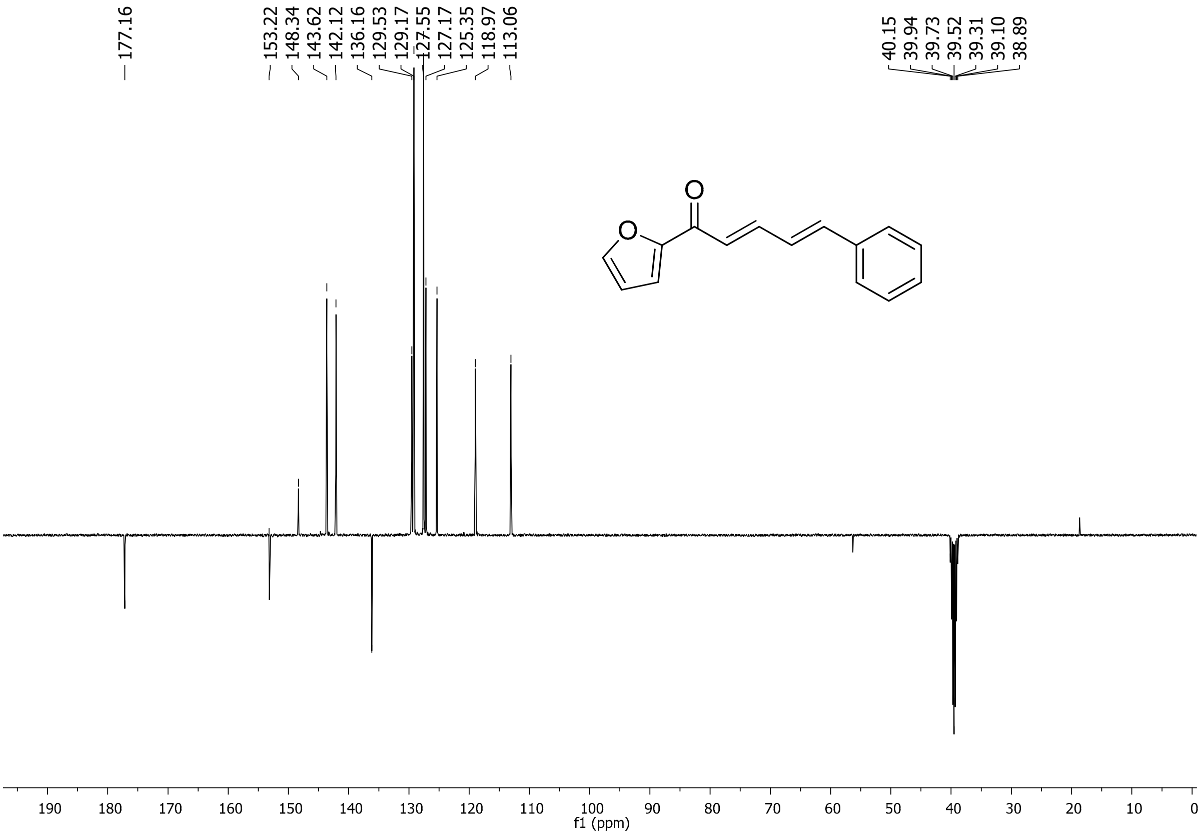

177.16
153.22
148.34
143.62
142.12
136.16
129.53
129.17
127.55
127.17
125.35
118.97
113.06
40.15
39.94
39.73
39.52
39.31
39.10
38.89
O
O
190
180
170
160
150
140
130
120
110
100
90
80
70
60
50
40
30
20
10
0
f1 (ppm)

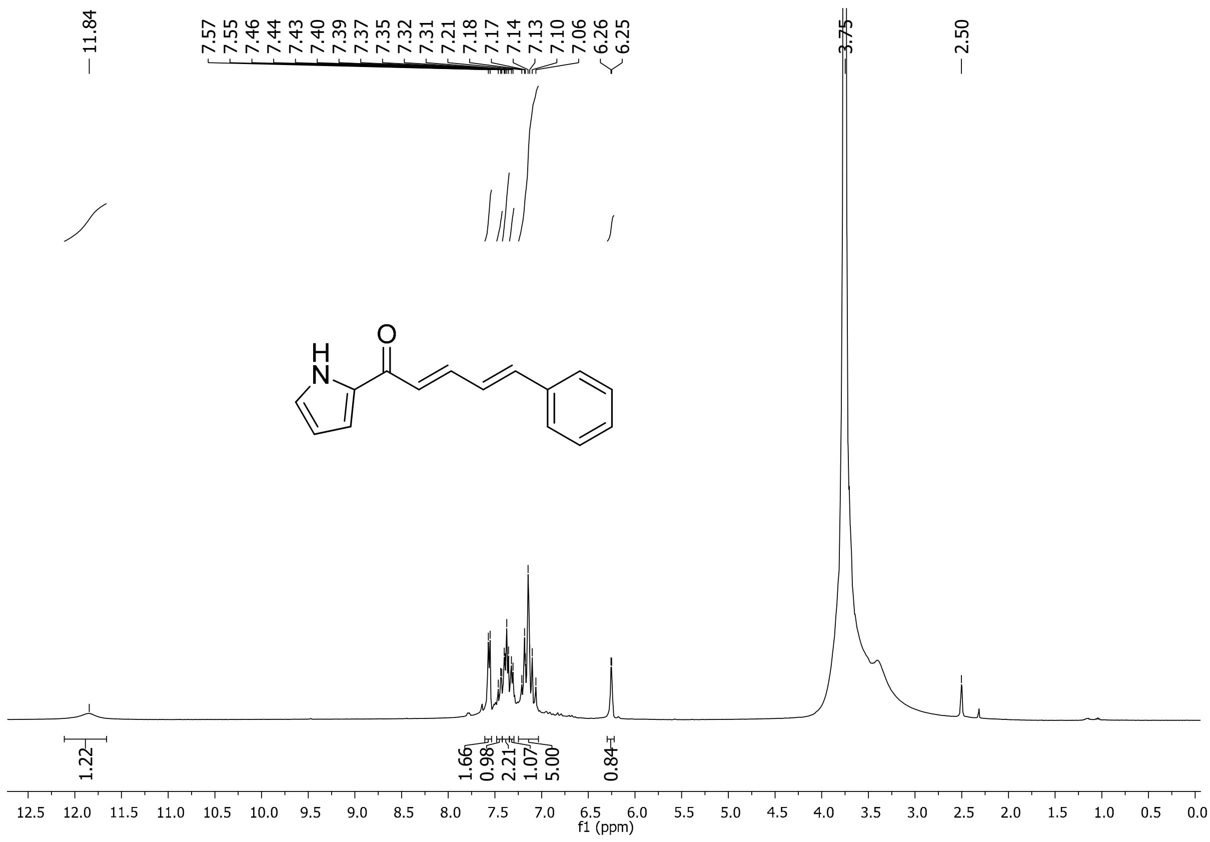

11.84
7.57
7.55
7.46
7.44
7.43
7.40
7.39
7.37
7.35
7.32
7.31
7.21
7.18
7.17
7.14
7.13
7.10
7.06
6.26
6.25
3.75
2.50
1.22
1.66
0.98
2.21
1.07
5.00
0.84
f1 (ppm)

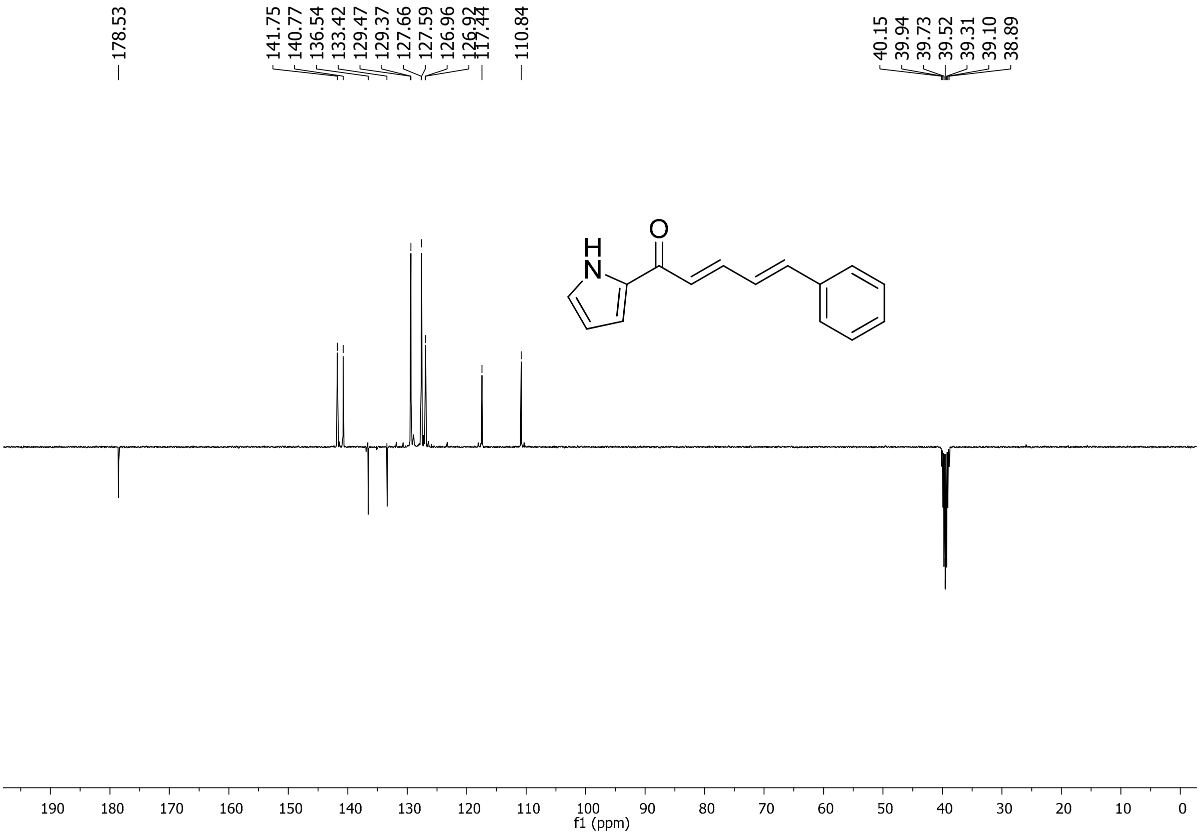
178.53
141.75
140.77
136.54
133.42
129.47
129.37
127.66
127.59
126.96
126.92
117.44
110.84
40.15
39.94
39.73
39.52
39.31
39.10
38.89
H
N
O
190
180
170
160
150
140
130
120
110
100
90
80
70
60
50
40
30
20
10
0
f1 (ppm)

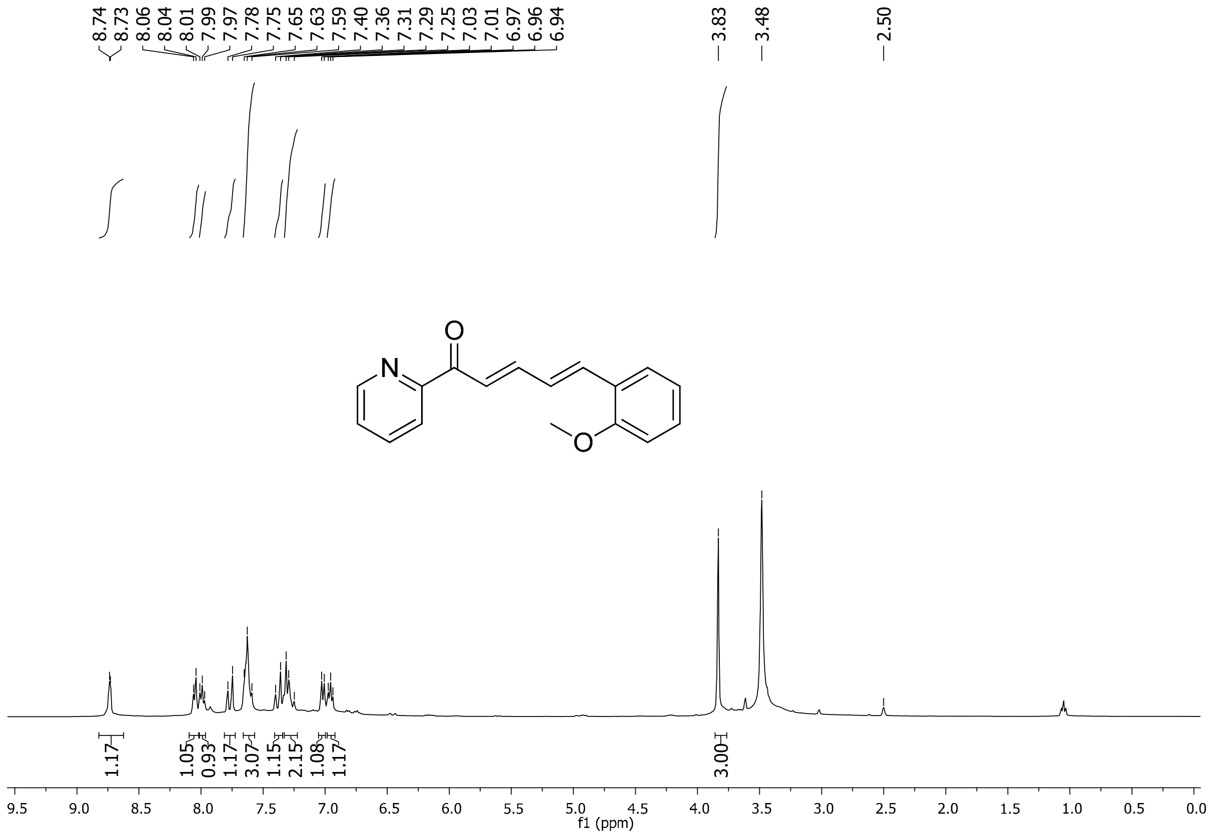

8.74
8.73
8.06
8.04
8.01
7.99
7.97
7.78
7.75
7.65
7.63
7.59
7.40
7.36
7.31
7.29
7.25
7.03
7.01
6.97
6.96
6.94
3.83
3.48
2.50
O
N
O
1.17
1.05
0.93
1.17
3.07
1.15
2.15
1.08
1.17
3.00
9.5
9.0
8.5
8.0
7.5
7.0
6.5
6.0
5.5
5.0
4.5
4.0
3.5
3.0
2.5
2.0
1.5
1.0
0.5
0.0
f1 (ppm)

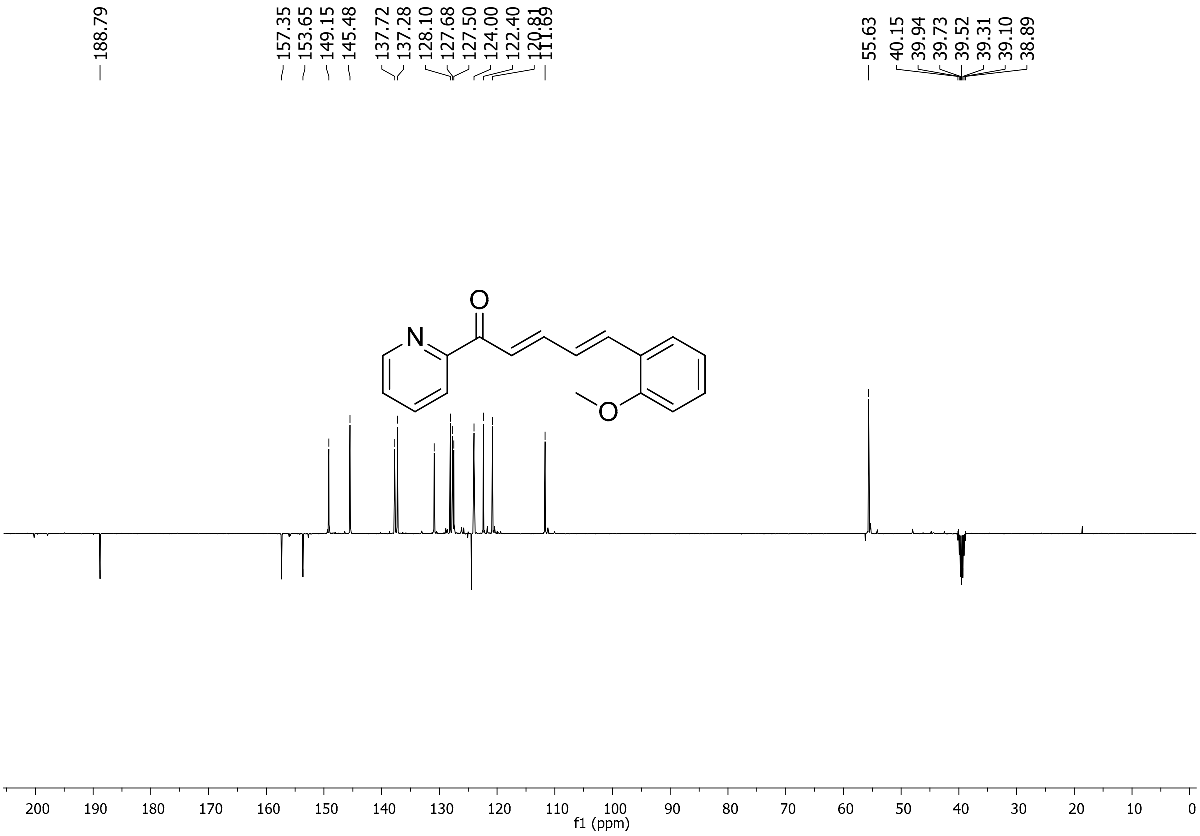
188.79
157.35
153.65
149.15
145.48
137.72
137.28
128.10
127.68
127.50
124.00
122.40
120.81
111.69
55.63
40.15
39.94
39.73
39.52
39.31
39.10
38.89
O
N
O
200
190
180
170
160
150
140
130
120
110
100
90
80
70
60
50
40
30
20
10
0
f1 (ppm)

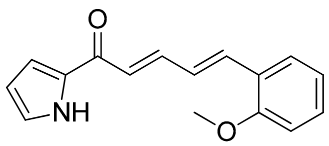
O
NH
O

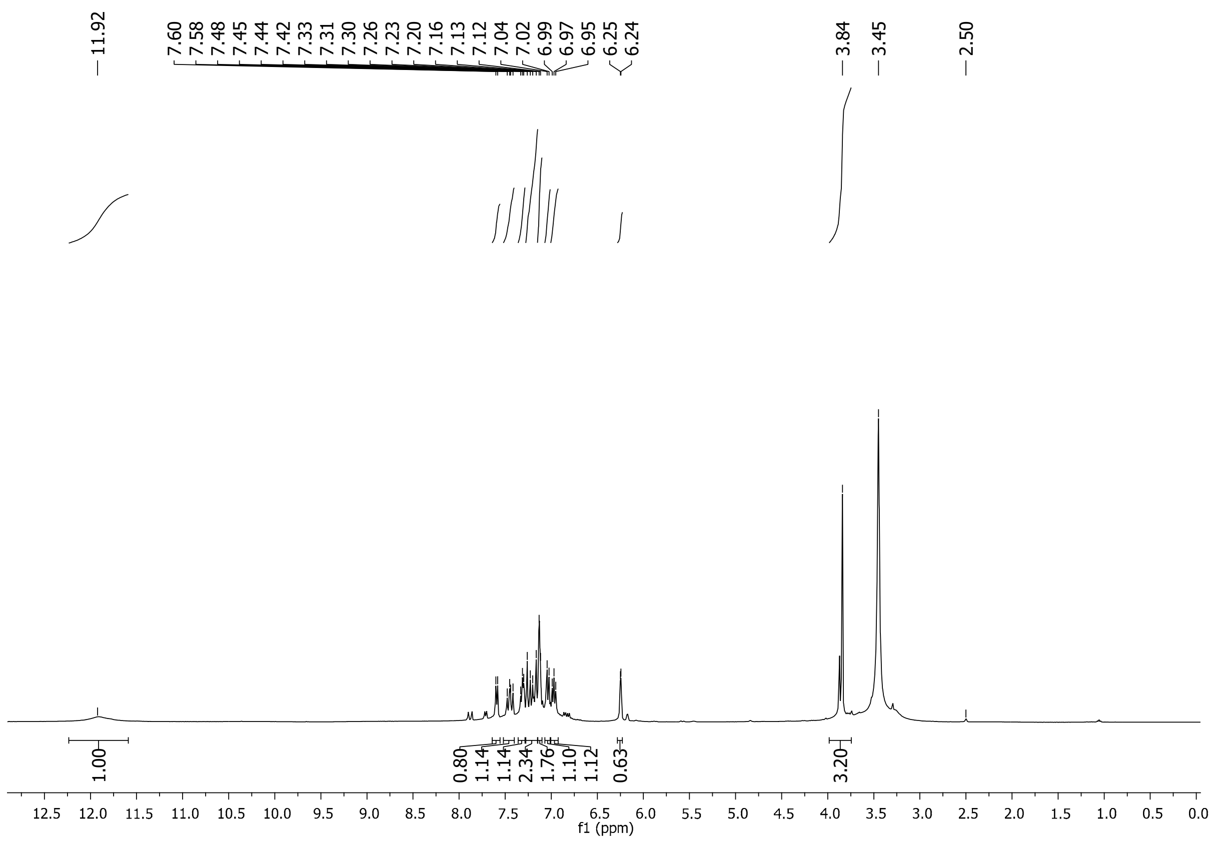
11.92
7.60
7.58
7.48
7.45
7.44
7.42
7.33
7.31
7.30
7.26
7.23
7.20
7.16
7.13
7.12
7.04
7.02
6.99
6.97
6.95
6.25
6.24
3.84
3.45
2.50
1.00
0.80
1.14
1.14
2.34
1.76
1.10
1.12
0.63
3.20
12.5 12.0 11.5 11.0 10.5 10.0 9.5 9.0 8.5 8.0 7.5 7.0 6.5 6.0 5.5 5.0 4.5 4.0 3.5 3.0 2.5 2.0 1.5 1.0 0.5 0.0
f1 (ppm)

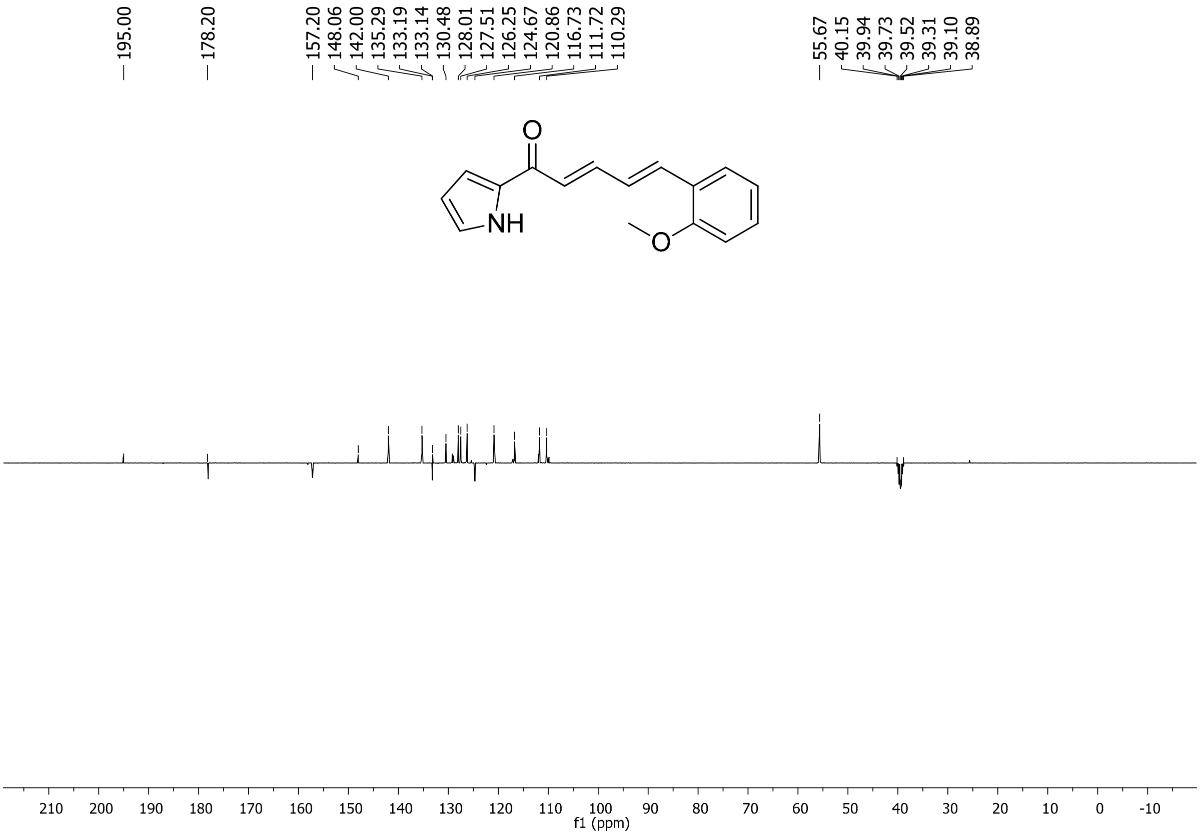
195.00
178.20
157.20
148.06
142.00
135.29
133.19
133.14
130.48
128.01
127.51
126.25
124.67
120.86
116.73
111.72
110.29
55.67
40.15
39.94
39.73
39.52
39.31
39.10
38.89
O
NH
O
210 200 190 180 170 160 150 140 130 120 110 100 90 80 70 60 50 40 30 20 10 0 -10
f1 (ppm)

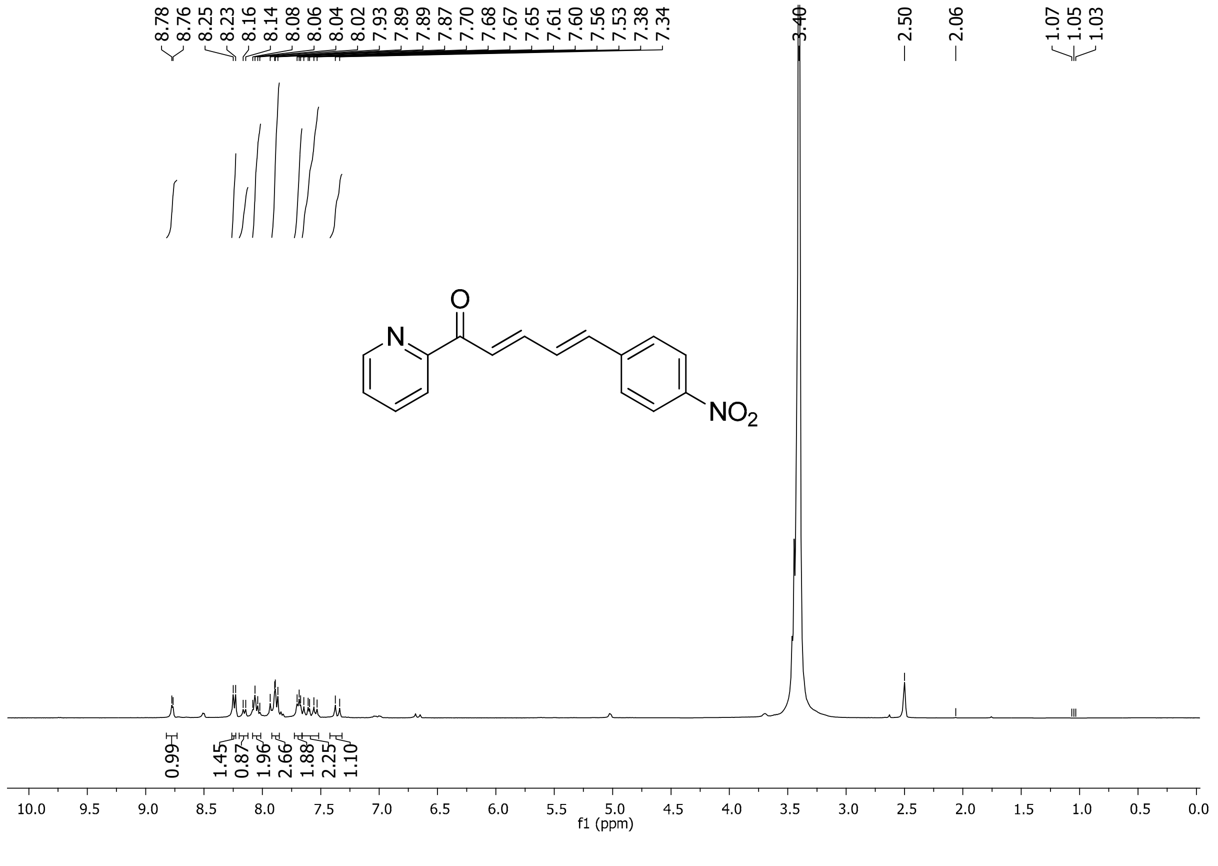

8.78
8.76
8.25
8.23
8.16
8.14
8.08
8.06
8.04
8.02
7.93
7.89
7.89
7.87
7.70
7.68
7.67
7.65
7.61
7.60
7.56
7.53
7.38
7.34
3.40
2.50
2.06
1.07
1.05
1.03
N
O
NO2
0.99
1.45
0.87
1.96
2.66
1.88
2.25
1.10
10.0
9.5
9.0
8.5
8.0
7.5
7.0
6.5
6.0
5.5
5.0
4.5
4.0
3.5
3.0
2.5
2.0
1.5
1.0
0.5
0.0
f1 (ppm)

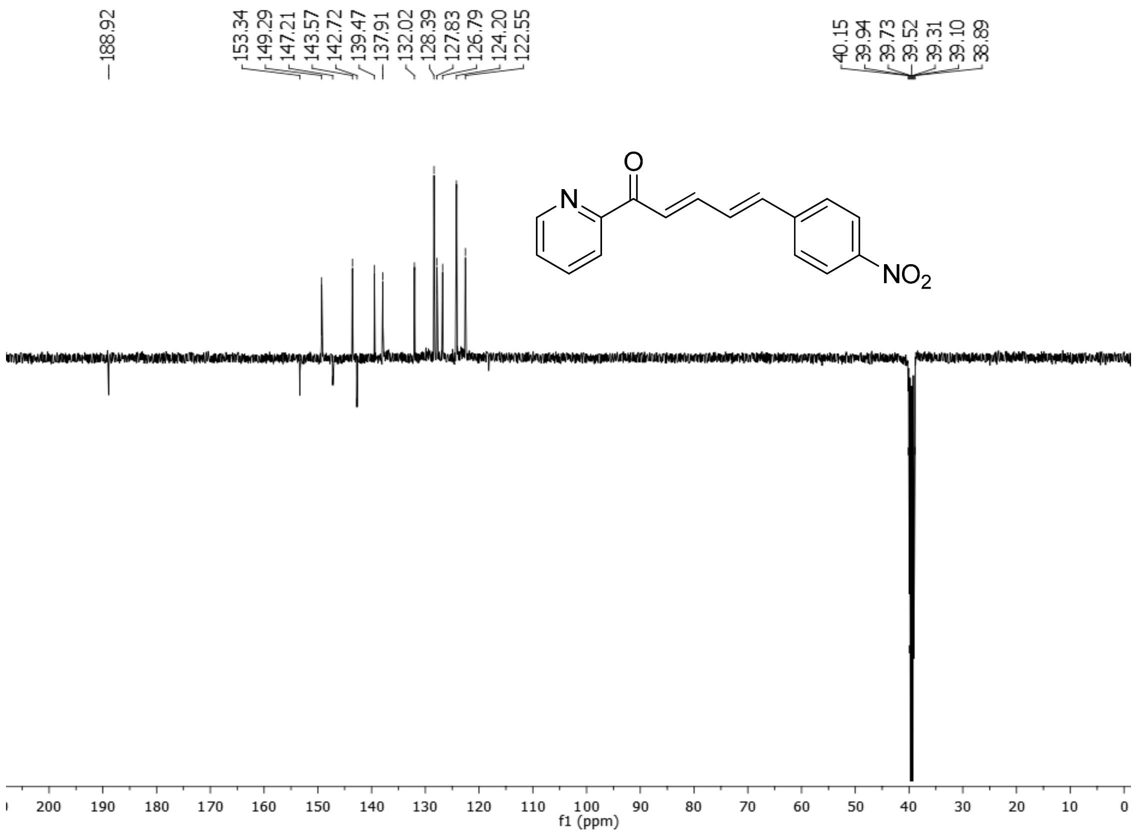

188.92
153.34
149.29
147.21
143.57
142.72
139.47
137.91
132.02
128.39
127.83
126.79
124.20
122.55
40.15
39.94
39.73
39.52
39.31
39.10
38.89
O
N
NO_2
200
190
180
170
160
150
140
130
120
110
100
90
80
70
60
50
40
30
20
10
0
f1 (ppm)

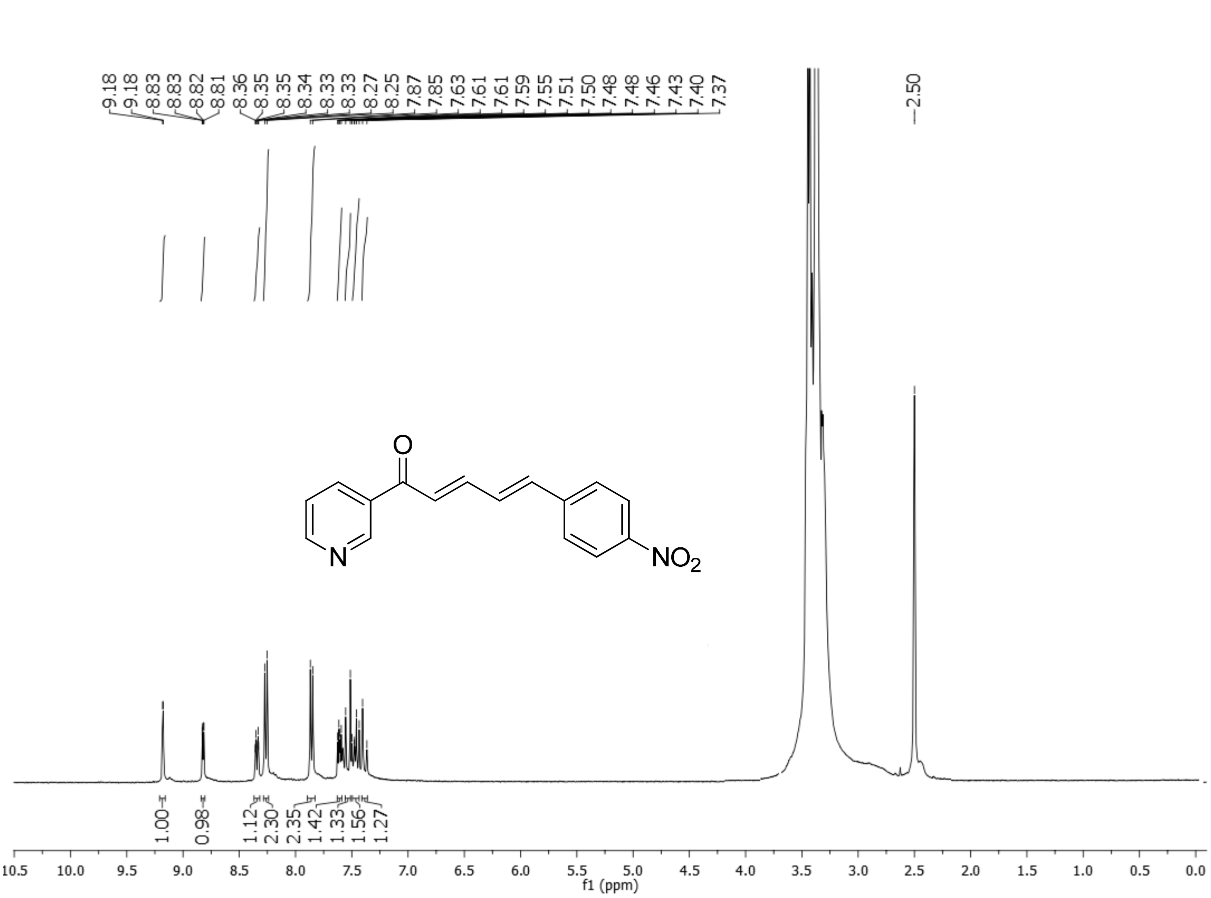

9.18
9.18
8.83
8.83
8.82
8.81
8.36
8.35
8.35
8.34
8.33
8.33
8.27
8.25
7.87
7.85
7.63
7.61
7.61
7.59
7.55
7.51
7.50
7.48
7.46
7.43
7.40
7.37
2.50
O
N
NO2
1.00
0.98
1.12
2.30
2.35
1.42
1.33
1.56
1.27
10.5
10.0
9.5
9.0
8.5
8.0
7.5
7.0
6.5
6.0
5.5
5.0
4.5
4.0
3.5
3.0
2.5
2.0
1.5
1.0
0.5
0.0
f1 (ppm)

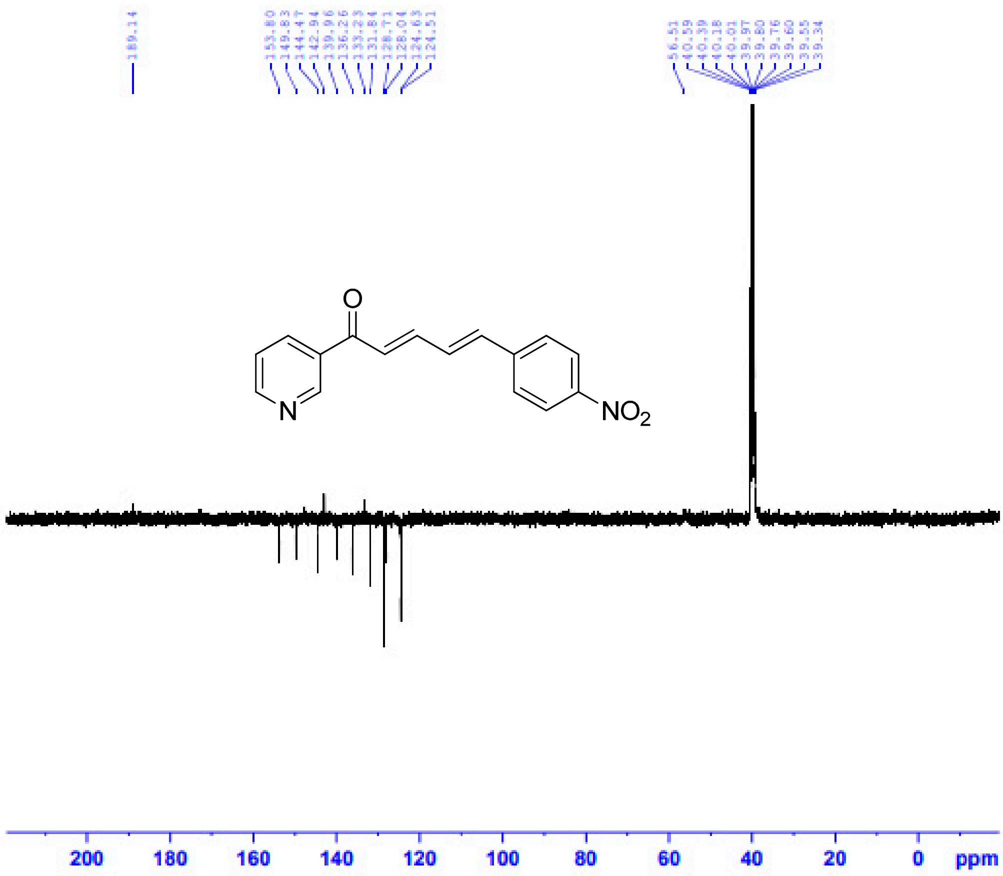

189.14
153.80
149.83
144.47
142.94
139.96
136.26
133.23
131.84
128.71
128.04
124.63
124.51
56.51
40.59
40.39
40.18
40.01
39.97
39.80
39.76
39.60
39.55
39.34
O
N
NO2
200
180
160
140
120
100
80
60
40
20
0
ppm

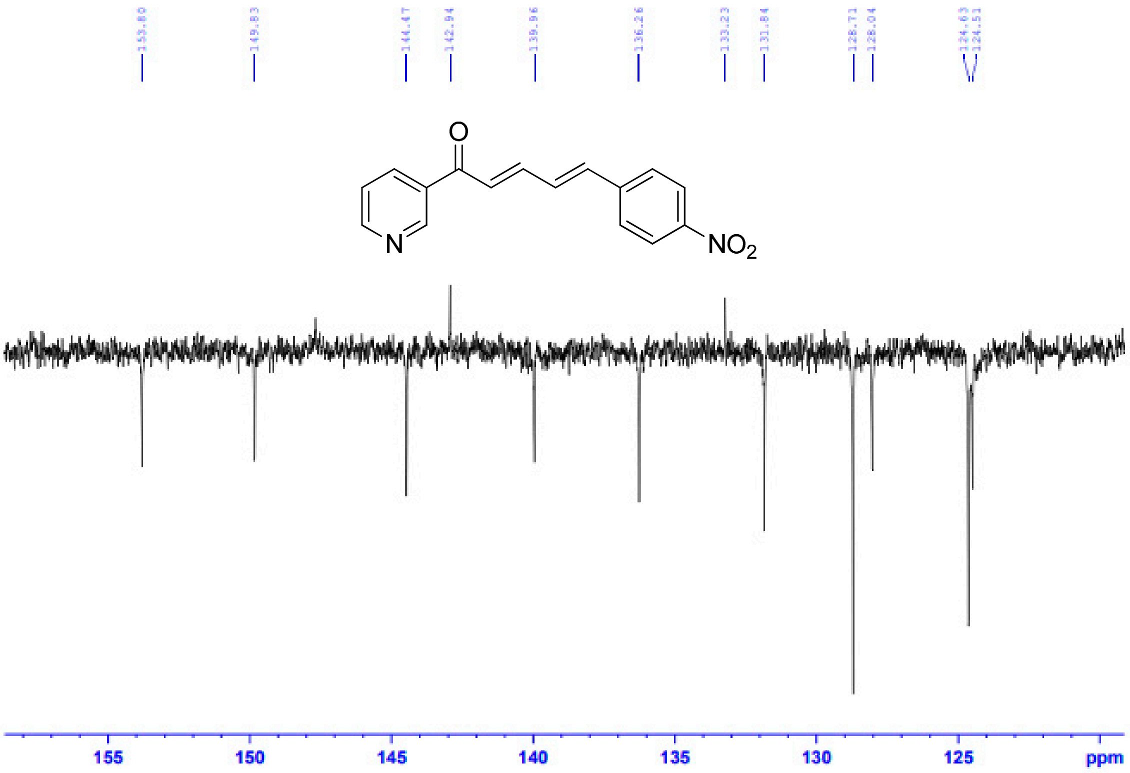
153.80
149.83
144.47
142.94
139.96
136.26
133.23
131.84
128.71
128.04
124.63
124.51
O
N
NO_2
155
150
145
140
135
130
125
ppm

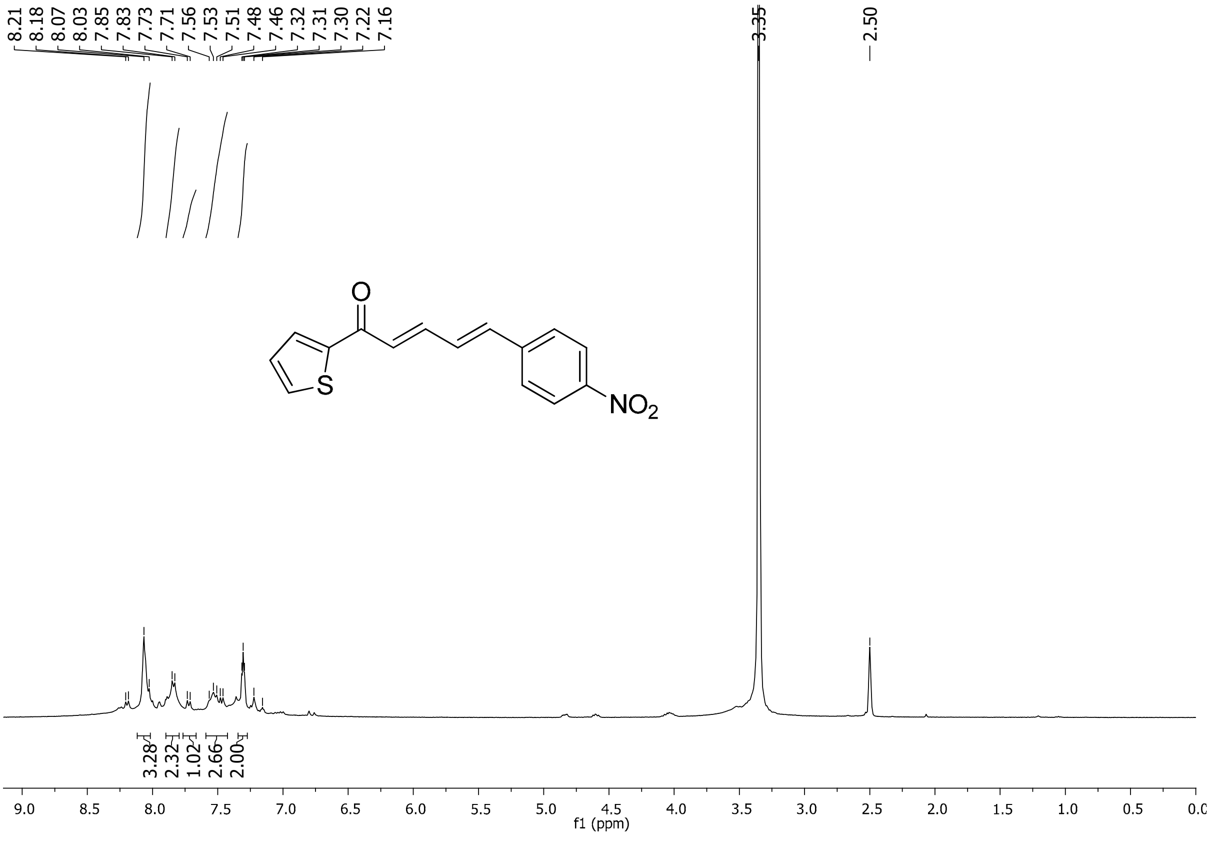

8.21
8.18
8.07
8.03
7.85
7.83
7.73
7.71
7.56
7.53
7.51
7.48
7.46
7.32
7.31
7.30
7.22
7.16
3.35
2.50
O
S
NO2
3.28
2.32
1.02
2.66
2.00
9.0
8.5
8.0
7.5
7.0
6.5
6.0
5.5
5.0
4.5
4.0
3.5
3.0
2.5
2.0
1.5
1.0
0.5
0.0
f1 (ppm)

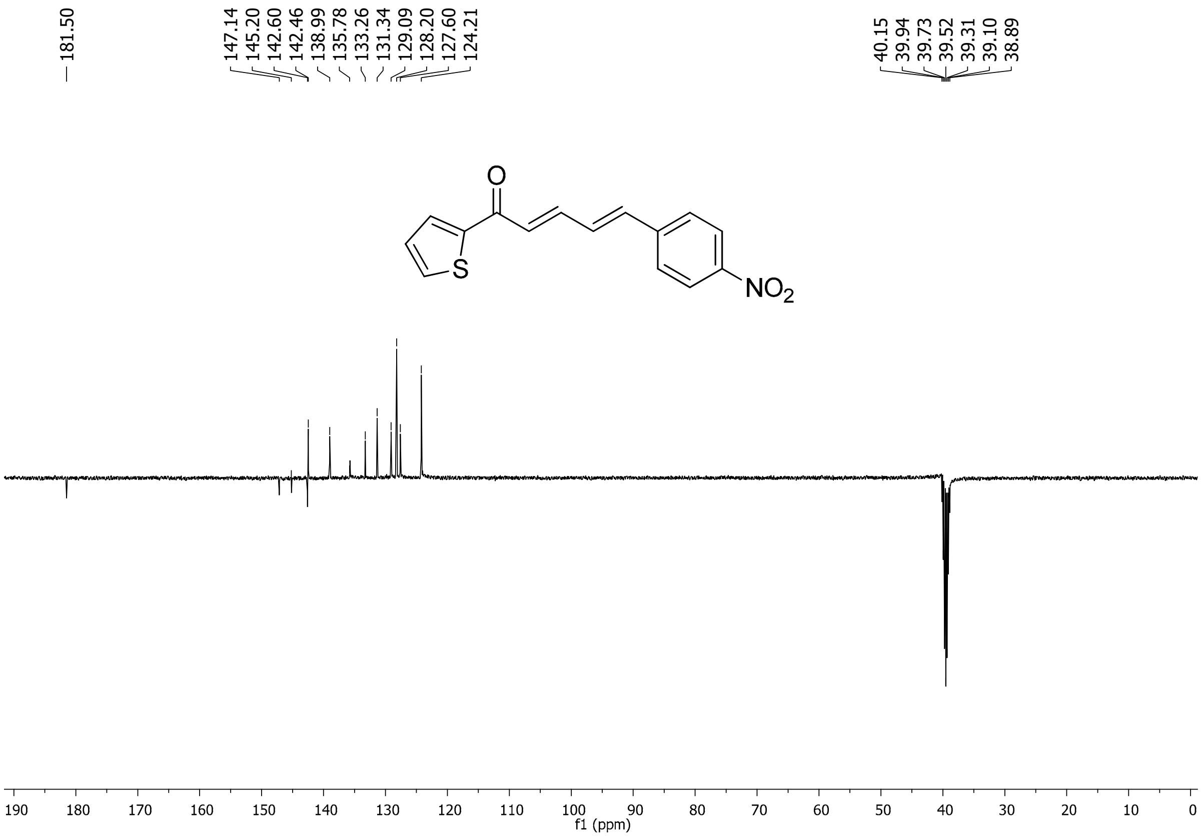

181.50
147.14
145.20
142.60
142.46
138.99
135.78
133.26
131.34
129.09
128.20
127.60
124.21
40.15
39.94
39.73
39.52
39.31
39.10
38.89
O
S
NO2
190 180 170 160 150 140 130 120 110 100 90 80 70 60 50 40 30 20 10 0
f1 (ppm)

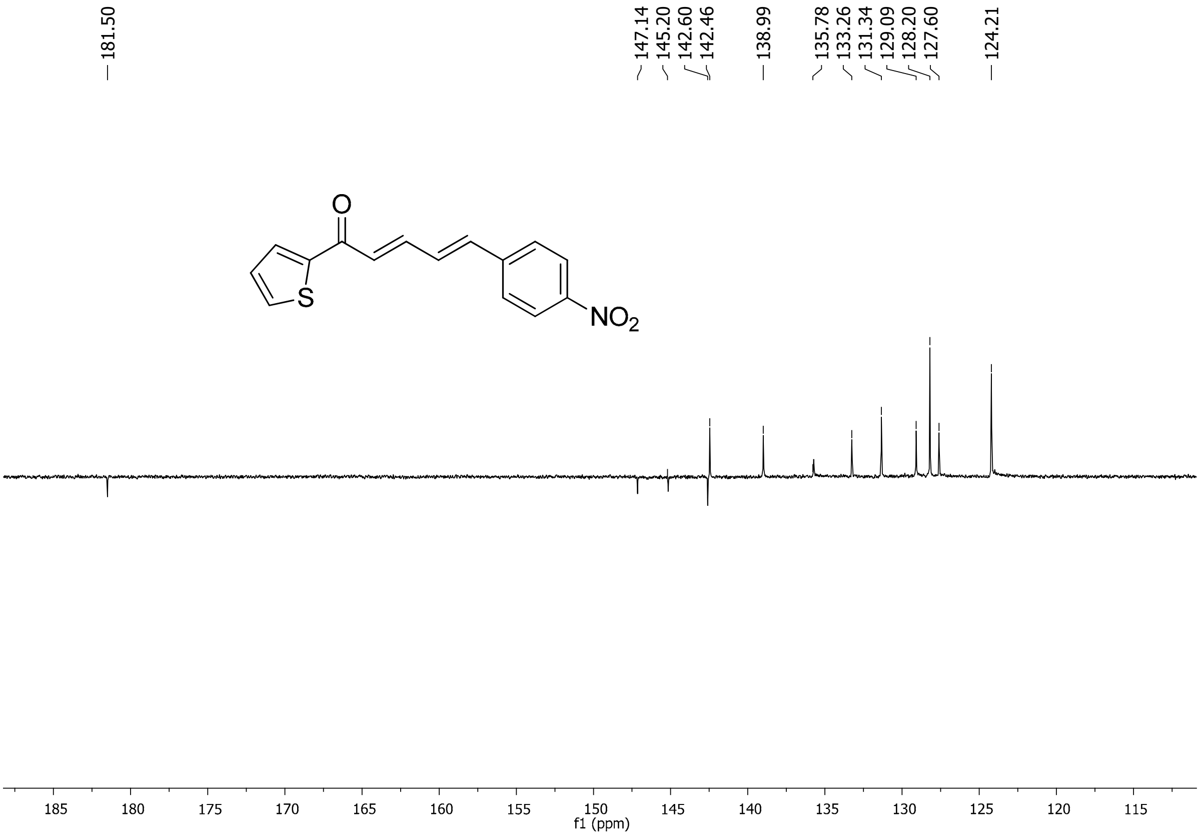
181.50
147.14
145.20
142.60
142.46
138.99
135.78
133.26
131.34
129.09
128.20
127.60
124.21
O
S
NO2
185
180
175
170
165
160
155
150
145
140
135
130
125
120
115
f1 (ppm)

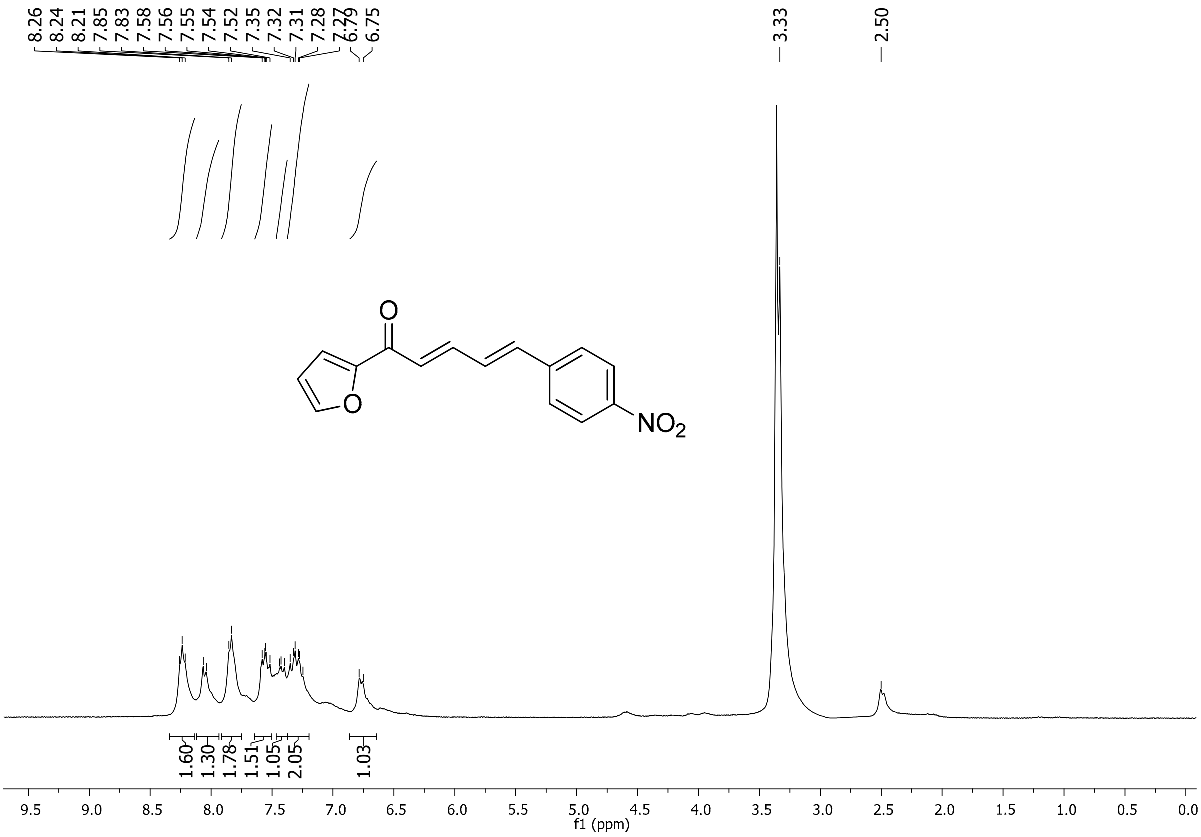
8.26
8.24
8.21
7.85
7.83
7.58
7.56
7.55
7.54
7.52
7.35
7.32
7.31
7.28
7.27
6.79
6.75
3.33
2.50
O
O
NO2
1.60
1.30
1.78
1.51
1.05
2.05
1.03
9.5
9.0
8.5
8.0
7.5
7.0
6.5
6.0
5.5
5.0
4.5
4.0
3.5
3.0
2.5
2.0
1.5
1.0
0.5
0.0
f1 (ppm)

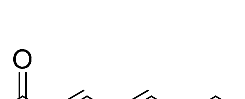
O

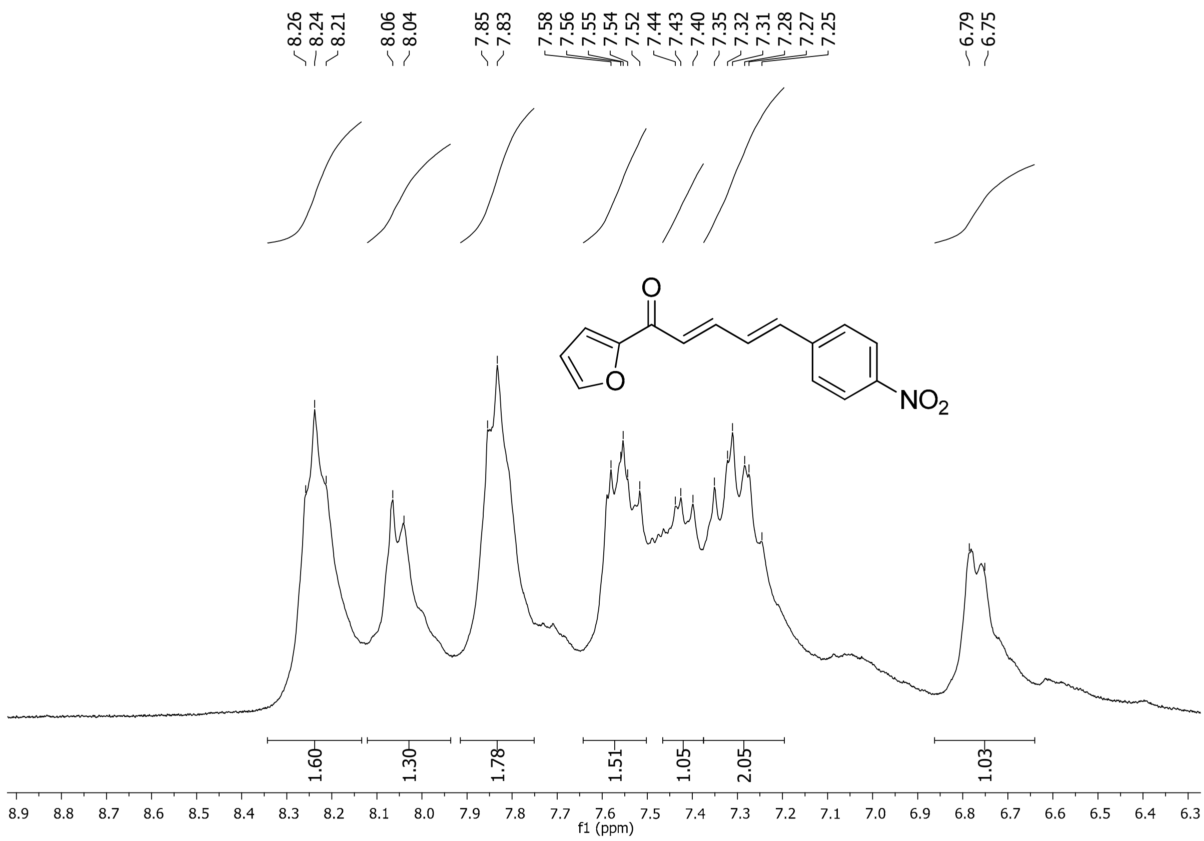

8.26
8.24
8.21
8.06
8.04
7.85
7.83
7.58
7.56
7.55
7.54
7.52
7.44
7.43
7.40
7.35
7.32
7.31
7.28
7.27
7.25
6.79
6.75
O
O
NO2
1.60
1.30
1.78
1.51
1.05
2.05
1.03
8.9 8.8 8.7 8.6 8.5 8.4 8.3 8.2 8.1 8.0 7.9 7.8 7.7 7.6 7.5 7.4 7.3 7.2 7.1 7.0 6.9 6.8 6.7 6.6 6.5 6.4 6.3
f1 (ppm)

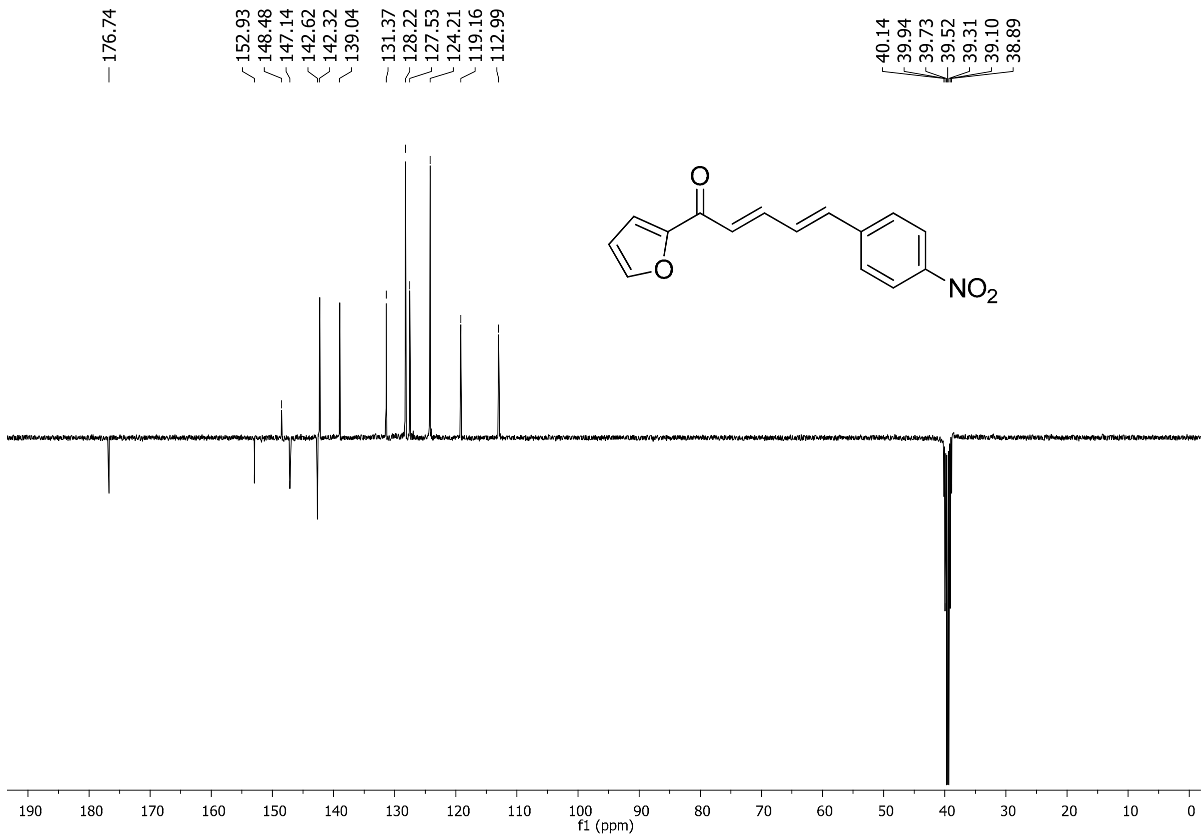

176.74
152.93
148.48
147.14
142.62
142.32
139.04
131.37
128.22
127.53
124.21
119.16
112.99
40.14
39.94
39.73
39.52
39.31
39.10
38.89
O
O
NO2
190 180 170 160 150 140 130 120 110 100 90 80 70 60 50 40 30 20 10 0
f1 (ppm)

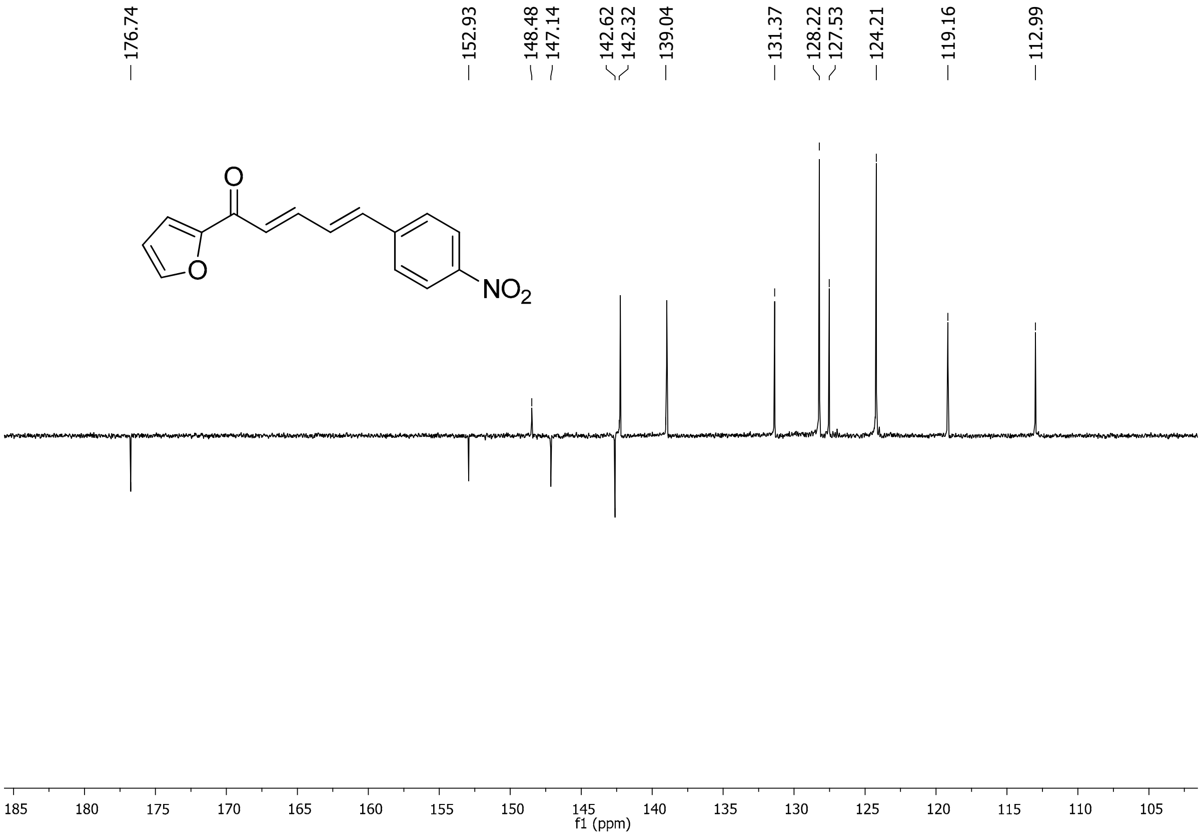
176.74
152.93
148.48
147.14
142.62
142.32
139.04
131.37
128.22
127.53
124.21
119.16
112.99
O
O
NO2
185
180
175
170
165
160
155
150
145
140
135
130
125
120
115
110
105
f1 (ppm)
